# Nuclear Magnetic Resonance Metabolomics of Symbioses between Bacterial Vaginosis-Associated Bacteria

**DOI:** 10.1128/msphere.00166-22

**Published:** 2022-05-02

**Authors:** Victoria Horrocks, Charlotte K. Hind, Matthew E. Wand, Paul-Enguerrand Fady, Joel Chan, Jade C. Hopkins, Georgina L. Houston, Rachel M. Tribe, J. Mark Sutton, A. James Mason

**Affiliations:** a Institute of Pharmaceutical Science, School of Cancer & Pharmaceutical Science, Faculty of Life Sciences and Medicine, King’s College London, London, United Kingdom; b Technology Development Group, Research and Evaluation Division, UK Health Security Agency, Salisbury, United Kingdom; c Department of Women and Children’s Health, School of Life Course and Population Sciences, Faculty of Life Sciences and Medicine, King’s College London, London, United Kingdom; Baylor College of Medicine

**Keywords:** bacterial vaginosis, spontaneous preterm birth, vaginal microbiome, *Prevotella bivia*, *Gardnerella vaginalis*, *Peptostreptococcus anaerobius*, *Atopobium vaginae*, *Mobiluncus curtisii*, *Gardnerella*, *Mobiluncus*, *Peptostreptococcus*, bacterial vaginosis, preterm birth

## Abstract

Bacterial vaginosis (BV) is a dysbiosis of the vaginal microbiome, characterized by low levels of lactobacilli and overgrowth of a diverse group of bacteria, associated with higher risk of a variety of infections, surgical complications, cancer, and preterm birth (PTB). Despite the lack of a consistently applicable etiology, *Prevotella* spp. are often associated with both BV and PTB, and *Pr. bivia* has known symbiotic relationships with both Peptostreptococcus anaerobius and Gardnerella vaginalis. Higher risk of PTB can also be predicted by a composite of metabolites linked to bacterial metabolism, but their specific bacterial source remains poorly understood. Here, we characterize diversity of metabolic strategies among BV-associated bacteria and lactobacilli and the symbiotic metabolic relationships between *Pr. bivia* and its partners and show how these influence the availability of metabolites associated with BV/PTB and/or pro- or anti-inflammatory immune responses. We confirm a commensal relationship between *Pe. anaerobius* and *Pr. bivia*, refining its mechanism, which sustains a substantial increase in acetate production. In contrast, the relationship between *Pr. bivia* and *G. vaginalis* strains, with sequence variant G2, is mutualistic, with outcome dependent on the metabolic strategy of the *G. vaginalis* strain. Taken together, our data show how knowledge of inter- and intraspecies metabolic diversity and the effects of symbiosis may refine our understanding of the mechanism and approach to risk prediction in BV and/or PTB.

**IMPORTANCE** Bacterial vaginosis (BV) is the most common vaginal infection for women of childbearing age. Although 50% of women with BV do not have any symptoms, it approximately doubles the risk of catching a sexually transmitted infection and also increases the risk of preterm delivery in pregnant women. Recent studies of the vaginal microbiota have suggested that variation between species in the same genus or between strains of the same species explain better or poorer outcomes or at least some coexistence patterns for bacteria of concern. We tested whether such variation is manifested in how vaginal bacteria grow in the laboratory and whether and how they may share nutrients. We then showed that this affected the overall cocktail of chemicals they produce, including bacterially derived chemicals that we have previously shown are linked to a higher risk of preterm delivery.

## INTRODUCTION

Bacterial vaginosis (BV) is regarded as a disruption of the lower genital tract microbiota with a shift from lactobacillus dominance to include a greater proportion of a range of species, including members of the genera *Gardnerella*, *Prevotella*, *Atopobium*, *Mobiluncus*, and *Peptostreptococcus* as well as *Sneathia*, *Leptotrichia*, and *Mycoplasma* and BV-associated bacterium 1 (BVAB1) to BVAB3 (1). Despite the lack of consistent etiology documented in women with BV, vaginal dysbiosis involving a plethora of species, irrespective of whether symptoms of BV are present, promotes local inflammation and is associated with a wide array of health problems (1).

A specific complication that may be related to BV is a 2-fold increased risk of spontaneous preterm birth (PTB) (2, 3). However, screening for asymptomatic BV in pregnancy in low-risk groups has not aided preterm birth prediction, and evidence is insufficient or conflicting even in studies of higher-risk groups (4). Nevertheless, numerous studies have pursued the association between the vaginal microbiome and PTB risk (5–16), including our own (15), and many of the species identified as associated with higher risk of PTB overlap those associated with BV.

Changes in microbiota composition are reflected in variations in bacterially derived metabolite profiles (11, 15, 17), which may have functional impact (18–21). Consistent with the microbiome studies, elevated vaginal lactate, which is the major product of the lactobacilli, and succinate have been found to be associated with term delivery (11), while elevated acetate was subsequently found to be higher in women who delivered preterm compared with term (17). A role for these metabolites in BV has also been considered (18, 21), with two studies agreeing that low lactate and high acetate and propionate are characteristic of BV (22, 23). Recently, we have shown that combining microbiome and metabolome into composite models has predictive value for preterm birth (15). A composite of metabolites that includes lactate and acetate but also aspartate, leucine, tyrosine, and betaine associated with risk of PTB at <37 weeks while risk of PTB at <34 weeks was identified by a composite of L. crispatus, L. acidophilus, glucose, and, again, aspartate.

Although multiple studies have identified *Prevotella* spp. as being associated with both BV and preterm birth (9, 12, 13, 15), their presence has not been found to be predictive of PTB (15). However, their residence within the vagina correlates with that of a number of other bacteria, including Gardnerella vaginalis (15, 16), and Prevotella bivia is known to enjoy symbiotic interactions with both Peptostreptococcus anaerobius and *G. vaginalis* (24–26). Two groups have found an association between preterm birth and *G. vaginalis* (7, 9, 16), but its presence alone does not predict PTB. However, there is reason to consider whether the substantial diversity of *G. vaginalis* affects the ability to establish its functional role(s) in both BV and preterm birth (27). Studies of microbial communities often sequence and quantify specific marker genes and cluster such sequences into operational taxonomic units (OTUs). Although such OTUs generally have been shown to have high levels of ecological consistency (28) and the approach remains popular and useful, there remains the possibility that functionally relevant differences in bacterial behavior are obscured by this approach. Indeed, in one study that confirmed an association between *G. vaginalis* and preterm birth, high-resolution statistical bioinformatics was used to detect nine unique *G. vaginalis* 16S rRNA sequence variants, and this revealed that only one of three *G. vaginalis* clades was responsible for the association of the genus with PTB (9). Strain-level profiling has also helped improve understanding of species cooccurrence profiles (16).

In addition, the role of the otherwise dominant lactobacilli may also be critical in defining PTB risk, with Lactobacillus crispatus dominance frequently associated with term delivery (9, 10, 13, 15, 16). The picture for *L. iners* is less clear. One study showed an association with PTB (10), but two subsequent studies found none (9, 15). Instead, they found frequent coexistence of *L. iners* with *G. vaginalis* (9), which contrasts with L. crispatus, where an exclusionary relationship with *G. vaginalis* is found (9, 16), or positive correlation with BV-associated bacteria, including *Pr. bivia* (15).

Given the valuable utility of the nuclear magnetic resonance (NMR) metabolomics approach for identifying risks associated with vaginal dysbiosis and predicting PTB and associations with differing microbiome states likely to have functional impact, there is an unmet need to understand bacterial contributions to the vaginal metabolome in more detail. To this end, we aimed to establish a mechanistic basis for a mutualistic symbiotic relationship between *Pr. bivia* and *G. vaginalis* and contrast this with the commensal relationship between *Pr. bivia* and *Pe. anaerobius.* We characterize the diverse metabolic strategies of a panel of *G. vaginalis* isolates and determine how this influences symbiosis with *Pr. bivia*. In addition, we compare metabolism across a panel of lactobacilli to highlight that variation in metabolic strategy is not limited to BV/sPTB-associated bacteria and that the metabolite background will likely vary according to microbiome community state type (CST) (5). The information provided by the present study suggests ways of refining prediction models that include metabolite data and gives insight into how bacterial metabolism and symbiosis influence each other, with implications for functional impact and clinical outcomes.

## RESULTS

To better understand the contribution of different bacteria to the vaginal metabolome in eubiosis and dysbiosis, a panel of lactobacilli and BV-associated isolates was assembled. Whole-genome sequencing of seven *G. vaginalis* strains included reference strains from the NCTC, and new isolates from vaginal swabs enables us to assign them to clades (9, 16, 29) or subgroup (30) and identifies genes for sialidase and vaginolysin (Table 1). Although expression was not tested, all isolates carry the genes coding for sialidase and vaginolysin. Strains KC1 and KC2 have type 1B vaginolysin, while the remainder have type 1A (31). Phylogeny analysis reveals six of the seven strains are members of clade 1/subgroup C/clade GV2a, corresponding to sequence variant G2 strains, which have been shown to drive observed associations with PTB (see Fig. S1 in the supplemental material) (9). The remaining isolate, KC1, is a member of clade 3/subgroup D/clade GV1b (Fig. S1). Tested for susceptibility to the main antibiotics used for BV, two isolates, KC1 and KC3, are found to be resistant to metronidazole and tinidazole. All isolates are sensitive to clindamycin and erythromycin.

**TABLE 1:** *G. vaginalis* strain characteristics^*a*^

Strain	Clade (29)/subgroup (30)/sequence variant (9)	Genome size (kb)	GC content (%)	MIC (μg/mL)			
				Clindamycin	Erythromycin	Metronidazole	Tinidazole
NCTC 10287	1/C/G2	1,663	41.3	0.015625	0.015625	4	2–4
NCTC 10915	1/C/G2	1,665	41.2	0.03125	0.015625	4	2
NCTC 11292	1/C/G2	1,659	41.3	0.03125	0.015625	4	2–4
KC1	3/D/G1	1,542	43.3	0.015625	0.015625	>256	128
KC2	1/C/G2	1,657	41.3	0.0625	0.015625	2	1–8
KC3	1/C/G2	1,733	41.1	0.0625	0.03125	16	64−128
KC4	1/C/G2	1,660	41.3	ng	0.0078125	4	ng

a All strains are positive for the genes encoding sialidase and vaginolysin. Concordant MICs are reported from three independently repeated experiments. ng, no growth.

10.1128/msphere.00166-22.1 FIG S1 Phylogenetic Tree showing similarity of *G. vaginalis* study isolates to other sequenced isolates. Neighbor-joining midpoint-rooted phylogenetic tree of the progressive alignment for the nucleotide sequences of *cpn60* (alignment length, 552 bp). The distance estimate is the similarity value subtracted from 1, where the similarity value is the number of similar nucleotides divided by the average sequence length to give a value between 0 and 1. Classification of groupings G1, G2, and G3 were taken from Callahan et al. (9). Six of seven strains from the present study (NCTC strains 11292, 10915 and 10287 and KC strains 2 to 4) cluster with sequence variants G2 while KC1 clusters with G1. Download FIG S1, PDF file, 0.08 MB. Copyright © 2022 Horrocks et al. 2022 Horrocks et al. https://creativecommons.org/licenses/by/4.0/ This content is distributed under the terms of the Creative Commons Attribution 4.0 International license.

### Overview of bacterial metabolism in BHI and identification of major metabolic strategies for BV-associated bacteria.

**FIG 1:**
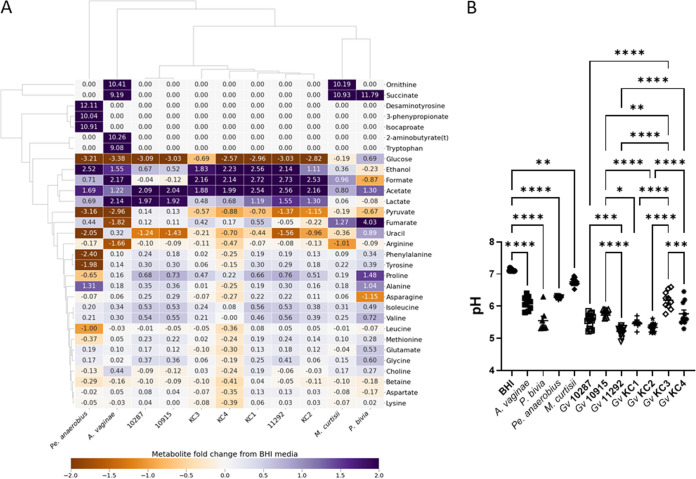
Diversity of bacterial vaginosis-associated bacterial metabolism when cultured in brain heart infusion. (A) The heatmap compares the metabolite fold change, relative to fresh BHI plus 5% horse serum, from ^1^H NMR of spent culture media and enables the major metabolites produced by each isolate and the key differences in metabolic strategy to be revealed. (B) The resulting acidification of the spent culture medium varies accordingly. Comparisons are shown between fresh BHI and the five non-*G. vaginalis* conditions and between each of the *G. vaginalis* strains, as determined by one-way analysis of variance (ANOVA) with Tukey’s correction for multiple comparisons. *, *P < *0.05; **, *P < *0.01; ***, *P < *0.001; ****, *P < *0.0001. All *G. vaginalis* strains acidify the media (*P < *0.0001).

**FIG 2:**
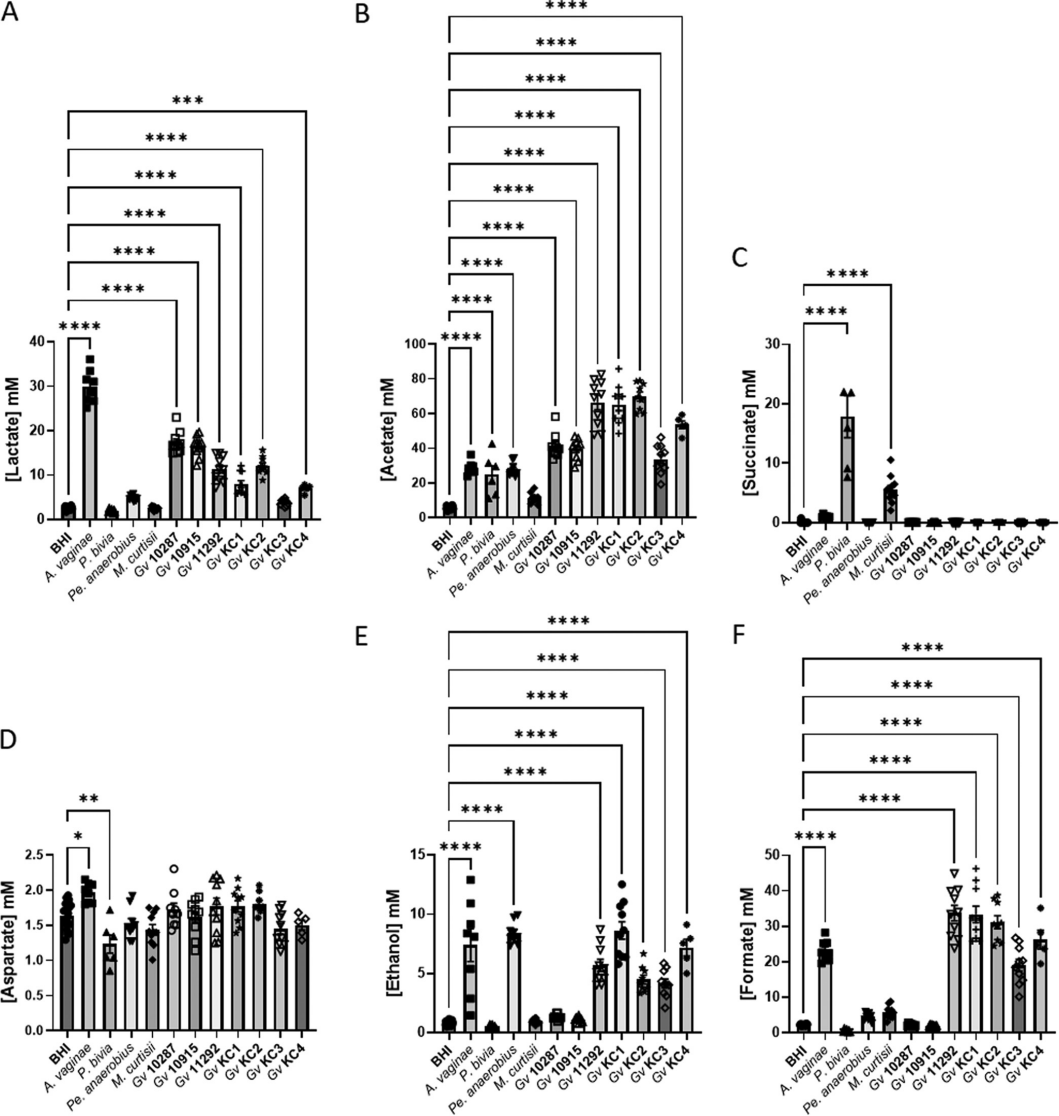
Production of organic acids, aspartate, and ethanol by bacterial vaginosis-associated bacteria in BHI. Comparisons are made between BHI plus 5% horse serum and each spent culture as determined by one-way ANOVA with Tukey correction for multiple comparisons for the main products of fermentation and/or those involved in anaerobic respiration. Comparisons for other metabolites are presented in Fig. S1. Only pairwise comparisons where *P *value is <0.05 are shown. *, *P < *0.05; **, *P < *0.01; ***, *P < *0.001; ****, *P < *0.0001.

While a chemically defined medium has been proposed for the culture of vaginal microflora (32), we found that growth of all seven *G. vaginalis* isolates in this medium is limited, *Pr. bivia* and *M. curtisii* exhibit very poor growth, and *Pe. anaerobius* was completely unable to grow. Pending development of a medium that mimics the secretions of the genital tract and also supports growth of all relevant bacterial species and strains, we opted to use BHI. BHI supports the growth of all isolates cultured in the present study, and analysis of BHI spent culture allows comparison of the overall metabolic strategy for each of the BV-associated bacteria but also comparison (Fig. 1) of the relative amounts of key metabolites that define the vaginal chemical environment in samples obtained from pregnant women by us (Table S1) or others (33). The NMR metabolomic approach clearly identifies the pyruvate and/or glucose fermentative strategies of *A. vaginae*, *Pe. anaerobius*, and the seven *G. vaginalis* isolates. The seven *G. vaginalis* isolates can be distinguished from each other and classified according to whether they use the bifid shunt (BS) alone, producing lactate and acetate from glucose (34), or mixed acid fermentation (MAF), producing lactate and acetate but also formate and ethanol and consuming pyruvate in addition to glucose (Fig. 1A and 2A, B, E, and F). *G. vaginalis* 10287 and 10915 are hence classified as using BS alone while the remainder use MAF.

10.1128/msphere.00166-22.8 TABLE S1 Comparison of metabolite concentrations in cervicovaginal fluid (CVF) swabs (from *n* = 96 pregnant participants) with corresponding values obtained from fresh and spent BHI following culture of BVAB or lactobacilli. The range and mean of CVF values include both women giving birth at term and preterm. Swabs concentrations correspond to the concentration once extracted into 750 μL phosphate-buffered saline (containing protease inhibitors and EDTA) collection buffer and hence is substantially diluted relative to the primary concentration in vaginal fluid but similar to previous work (33). Mean [lactate] in vaginal fluid has been measured at 87.7 mM, giving a 24-fold dilution during swab collection (52). Download Table S1, PDF file, 0.1 MB. Copyright © 2022 Horrocks et al. 2022 Horrocks et al. https://creativecommons.org/licenses/by/4.0/ This content is distributed under the terms of the Creative Commons Attribution 4.0 International license.

*M. curtisii* is known to be capable of using trimethylamine oxide (TMAO) as an electron donor for anaerobic respiration, producing trimethylamine (TMA) (35). In BHI it also conducts anaerobic respiration, but the production of succinate (Fig. 1A and 2C) is suggestive of fumarate acting as the electron donor in place of TMAO, which is absent. *M. curtisii* is known also to consume arginine to produce ornithine, citrulline, and ammonia (36), and both it and *A. vaginae* do this also in BHI (Fig. 1A and Fig. S2E and T). *Pr. bivia* characteristically also produces succinate via anaerobic respiration but also ferments glucose to acetate (37), and this is observed in BHI alongside avid consumption of asparagine (Fig. 1A and Fig. S2B to D). *Pr. bivia* notably excretes a variety of metabolites that are not produced at the same levels or at all and are often consumed by the other BV-associated bacteria. These include succinate, fumarate, alanine, glutamate, glycine, methionine, phenylalanine, proline, valine, and uracil (Fig. 1A and Fig. S2C, H, J, L, M to P, R, and S). A little threonine is produced by four of the seven *G. vaginalis* isolates and *Pr. bivia*, while it is avidly consumed by *A. vaginae* and *Pe. anaerobius* (Fig. 1A and Fig. S2U). The result of these differing metabolic strategies is, in every case, an acidification of the spent BHI culture, but this is relatively modest for *M. curtisii*, *Pe. anaerobius*, and *A. vaginae* compared with that observed for the seven *G. vaginalis* strains and *P. bivia* (Fig. 1B).

10.1128/msphere.00166-22.2 FIG S2 Univariate analysis of spent BHI cultures of bacterial vaginosis-associated bacteria. Comparisons are made between BHI and each spent culture as determined by one-way ANOVA with Tukey correction for multiple comparisons for the main products of fermentation and/or those involved in anaerobic respiration. Only pairwise comparisons where *P *value is <0.05 are shown. *, *P < *0.05; **, *P < *0.01; ***, *P < *0.001; ****, *P < *0.0001. Download FIG S2, PDF file, 0.3 MB. Copyright © 2022 Horrocks et al. 2022 Horrocks et al. https://creativecommons.org/licenses/by/4.0/ This content is distributed under the terms of the Creative Commons Attribution 4.0 International license.

**FIG 3:**
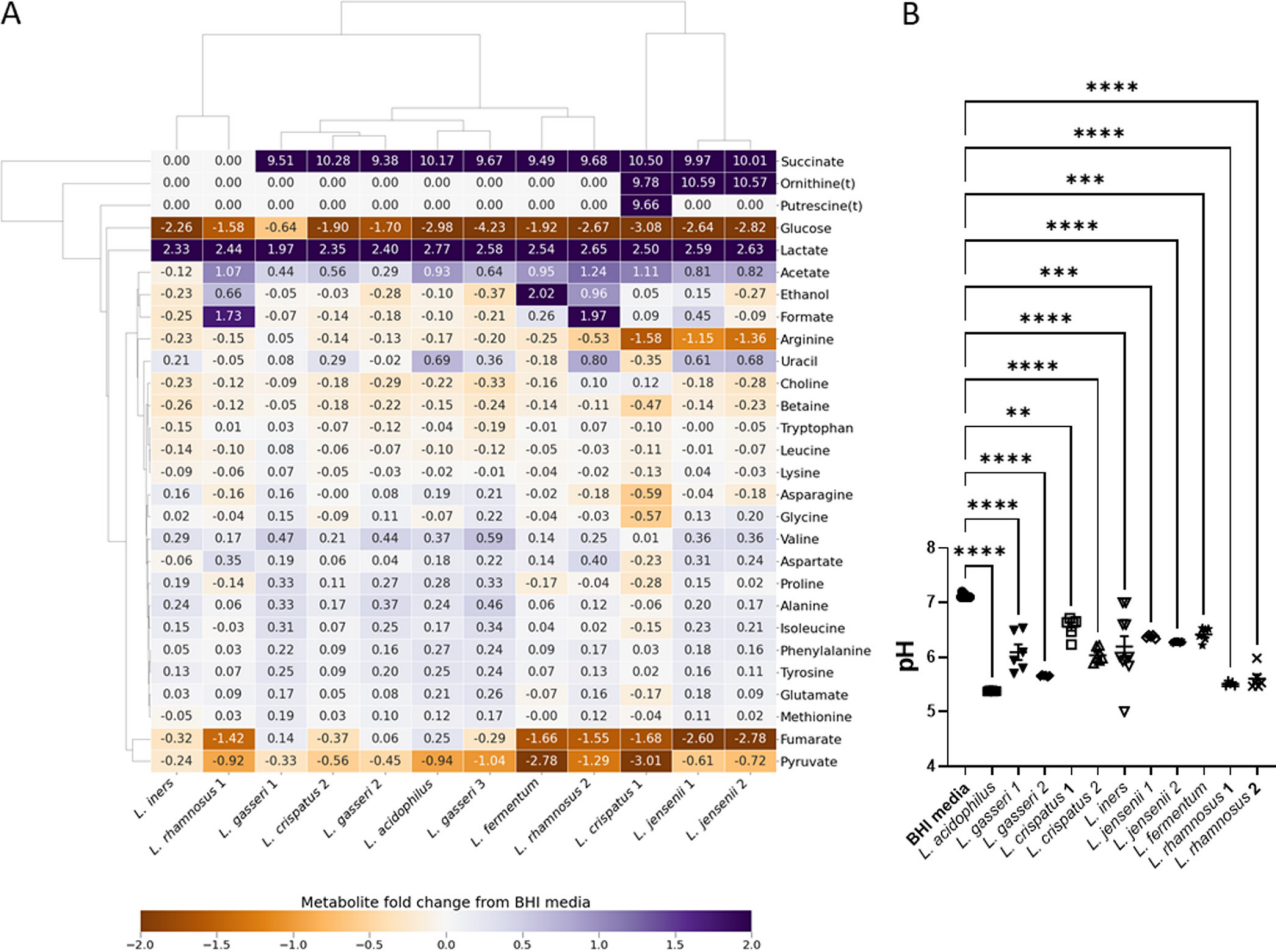
Diversity of *Lactobacillus* species metabolism when cultured in brain heart infusion. (A) The heatmap compares the metabolite concentration fold change, relative to fresh BHI plus 5% horse serum, from ^1^H NMR determined concentrations in spent culture media and enables the major metabolites produced by each isolate and the key differences in metabolic strategy to be revealed. (B) The resulting acidification of the spent culture media accordingly varies. Comparisons are shown between fresh BHI and each spent culture, as determined by one-way ANOVA with Tukey correction for multiple comparisons. *, *P < *0.05; **, *P < *0.01; ***, *P < *0.001; ****, *P < *0.0001.

**FIG 4:**
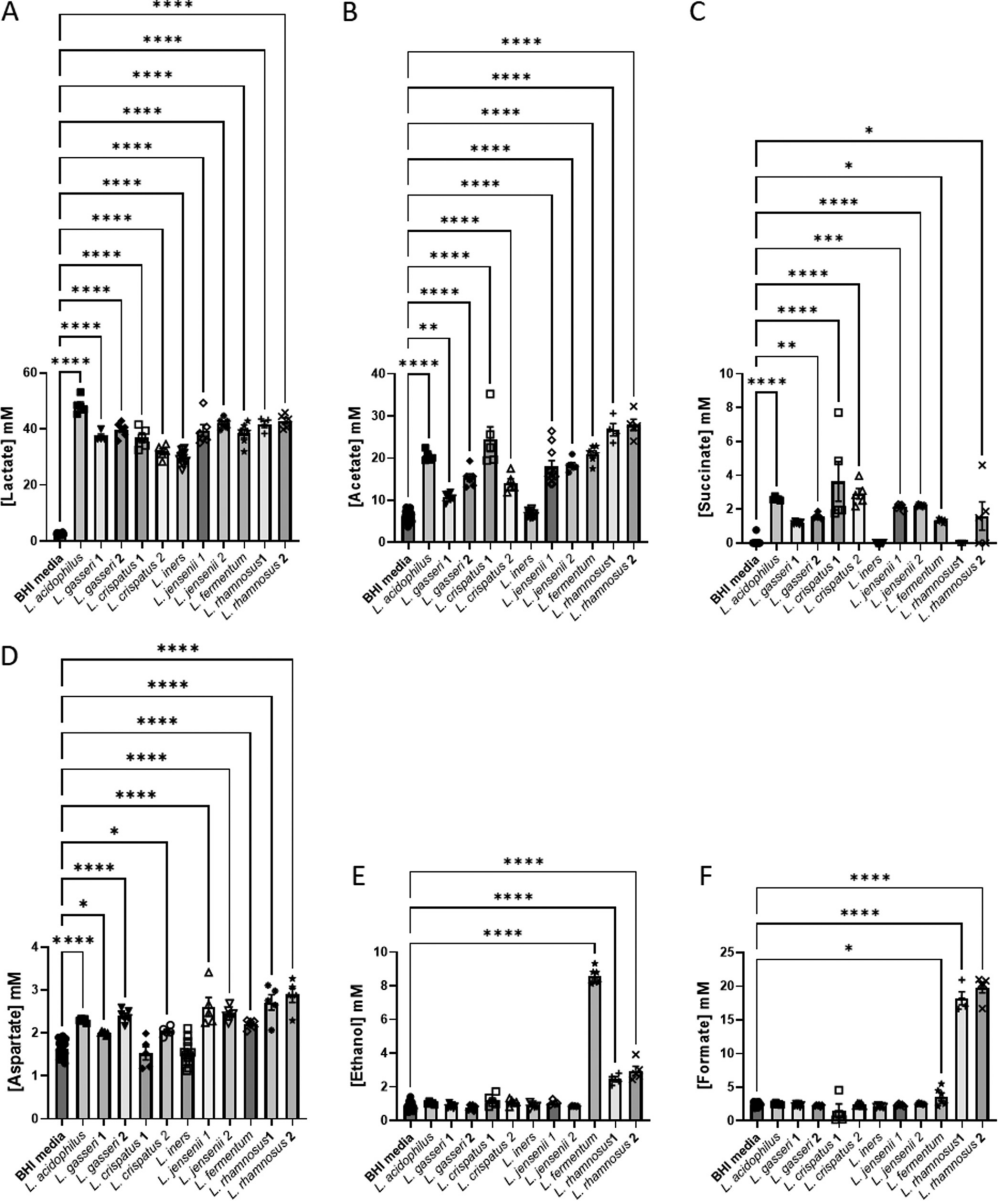
Production of organic acids, aspartate, and ethanol by *Lactobacillus* spp. in BHI. Comparisons are made between BHI plus 5% horse serum and each spent culture as determined by one-way ANOVA with Tukey correction for multiple comparisons for the main products of fermentation and/or those involved in anaerobic respiration. Comparisons for other metabolites are presented in Fig. S1. Only pairwise comparisons where *P *value is <0.05 are shown. *, *P < *0.05; **, *P < *0.01; ***, *P < *0.001; ****, *P < *0.0001.

Considering the lactobacilli, four species are considered obligate homofermentative (L. acidophilus, L. crispatus, L. gasseri, and *L. iners*) using the Embden-Meyerhof-Parnas (EMP) pathway to make lactate (both d-lactate and l-lactate, with the exception of *L. iners*, which makes only l-lactate), two species are considered facultative heterofermentative, making lactate (l-lactate for L. rhamnosus and d-lactate for L. jensenii) and acetate, and one species, *L. fermentum*, is obligate heterofermentative, producing lactate, acetate, and ethanol as well as CO_2_ (38). The present NMR results are consistent with this, with all lactobacilli producing lactate (Fig. 3 and 4), only *L. fermentum* producing substantial quantities of ethanol (Fig. 3A and 4E), and only L. rhamnosus producing substantial amounts of formate (Fig. 3A and 4F). Consistent with genome sequence studies, which showed a lack of enzymes to produce acetate (39, 40), *L. iners* is the only *Lactobacillus* in this study that does not produce any acetate in BHI; acetate production by the other lactobacilli varies considerably (Fig. 3A and 4B).

The lactobacilli can be further distinguished, notably at strain level for L. crispatus, by differing consumption of pyruvate, asparagine, arginine, glycine, lysine, and proline (Fig. 3A and Fig. S3B, D, J, K, M, P, and R) and production of alanine, valine, isoleucine, and uracil (Fig. 3A and Fig. S3K, S, T, and U). Acidification of the spent culture medium is likely limited by the relatively low glucose concentration in BHI, but the greatest acidification is achieved by L. acidophilus (significantly more than all except L. crispatus 2), which also produces more lactate than any of the other strains (*P < *0.05) (Fig. 3B and 4A).

10.1128/msphere.00166-22.3 FIG S3 Univariate analysis of BHI spent cultures of lactobacilli. Comparisons are made between BHI and each spent culture as determined by one-way ANOVA with Tukey correction for multiple comparisons for the main products of fermentation and/or those involved in anaerobic respiration. Only pairwise comparisons where *P *value is <0.05 are shown. *, *P < *0.05; **, *P < *0.01; ***, *P < *0.001; ****, *P < *0.0001. Download FIG S3, PDF file, 0.2 MB. Copyright © 2022 Horrocks et al. 2022 Horrocks et al. https://creativecommons.org/licenses/by/4.0/ This content is distributed under the terms of the Creative Commons Attribution 4.0 International license.

We have shown previously that lower lactate and higher acetate were associated with increased risk of PTB at <37 weeks (odds ratios, 0.432 and 1.610) (15). As expected, and again despite the relatively low concentration of glucose in BHI, the lactobacilli produce a final lactate concentration of between 30 and 45 mM in spent BHI culture (Fig. 4A), which substantially exceed production by BV-associated bacteria (Fig. 2A). Of note, however, is that *A. vaginae* spent culture is enriched with around 27 mM lactate, and the two BS *G. vaginalis* strains produce substantially more lactate than the five MAF *G. vaginalis* strains (*P < *0.0001) (Fig. 2A). Except for *L. iners*, acetate is produced by lactobacilli in BHI to achieve final concentrations ranging from 5 mM to 21 mM (Fig. 4). Similar levels of acetate production are achieved by *A. vaginae*, *Pr. bivia*, and *Pe. anaerobius*, but this is dwarfed by production by *G. vaginalis* with BS strains attaining 35 mM and MAF strains as much as 65 mM. Although succinate secretion is a hallmark of anaerobic respiration and concentrations of nearly 18 mM are achieved in *Pr. bivia* spent culture (Fig. 2C), small amounts of this dicarboxylate (1 to 4 mM) are also detected in all lactobacillus spent cultures with the exception again of *L. iners* and also L. rhamnosus 1 (Fig. 4C).

Higher aspartate has previously been associated with increased risk of PTB at <37 and <34 weeks (odds ratios, 1.675 and 1.768) (15). Seven of the nine lactobacillus strains produce this, but this is very modest with spent culture enriched by a maximum of 1.2 mM aspartate (Fig. 4D). In monoculture, none of the *G. vaginalis* strains produce aspartate, but modest amounts are produced by *A. vaginae*, and it is consumed by *Pr. bivia* (Fig. 2D). We have reported higher glucose associated with increased risk of PTB at <34 weeks (odds ratio, 1.269) (15). Almost all glucose in BHI is consumed by both lactobacilli and BV-associated bacteria, with the exception of *M. curtisii* (Fig. 1A and 3A and Fig. S2A and S3A). *G. vaginalis* KC3 is somewhat fastidious and did not consume all glucose in this first study (Fig. 1A and Fig. S2A), but in the coculture experiments described below it grew well, and its consumption matched that of the other *G. vaginalis* strains (see Fig. 6A and Fig. S6A). In contrast, pyruvate available in BHI is not universally consumed (Fig. 1A and 3A and Fig. S2B and S3B). *A. vaginae*, *Pe. anaerobius*, L. crispatus 1, and *L. fermentum* consume all pyruvate available, while the remaining lactobacilli and BV-associated bacteria, except for *G. vaginalis* 10287 and 10915, consume some but not all. *G. vaginalis* 10287 and 10915 secrete modest amounts of pyruvate into the spent culture (*P < *0.05).

10.1128/msphere.00166-22.6 FIG S6 Univariate analysis of spent BHI metabolite concentrations for *Pr. bivia*/*G. vaginalis* mono- and coculture. Comparisons are shown between each coculture and the corresponding monocultures as determined by one-way ANOVA with Tukey correction for multiple comparisons. Only *P* values <0.05 are shown. *, *P < *0.05; **, *P < *0.01; ***, *P < *0.001; ****, *P < *0.0001. Download FIG S6, PDF file, 0.3 MB. Copyright © 2022 Horrocks et al. 2022 Horrocks et al. https://creativecommons.org/licenses/by/4.0/ This content is distributed under the terms of the Creative Commons Attribution 4.0 International license.

Higher leucine and betaine and lower tyrosine have also been associated with increased risk of PTB at <37 weeks (odds ratios, 3.118, 1.365, and 0.023) (15). None of the lactobacilli or BV-associated bacteria in the present study produce leucine when cultured in BHI, although it is avidly consumed by *Pe. anaerobius* (Fig. 1A and 3A and Fig. S2G and S3G). Tyrosine is produced in modest amounts by six of the lactobacilli isolates, most notably by L. acidophilus, L. gasseri 1 and 2, and most *G. vaginalis* strains as well as *A. vaginae*, *Pr. bivia*, and *M. curtisii* (Fig. 1A and 3A and Fig. S2C). It is consumed avidly by *Pe. anaerobius* (Fig. 1A and Fig. S2C). With the exception of *Pe. anaerobius*, L. crispatus 1, and *L. iners*, where there is modest consumption, the concentration of betaine does not change in the spent culture of either the lactobacilli or the BV-associated bacteria (Fig. 1A and 3A and Fig. S2E and S3H). Similarly, with the exception of *A. vaginae*, changes in choline concentrations are minimal (Fig. 1A and 3A and Fig. S2F and S3F).

### Symbiosis between *Pr. bivia* and *Pe. anaerobius* influences production of key PTB markers.

**FIG 5:**
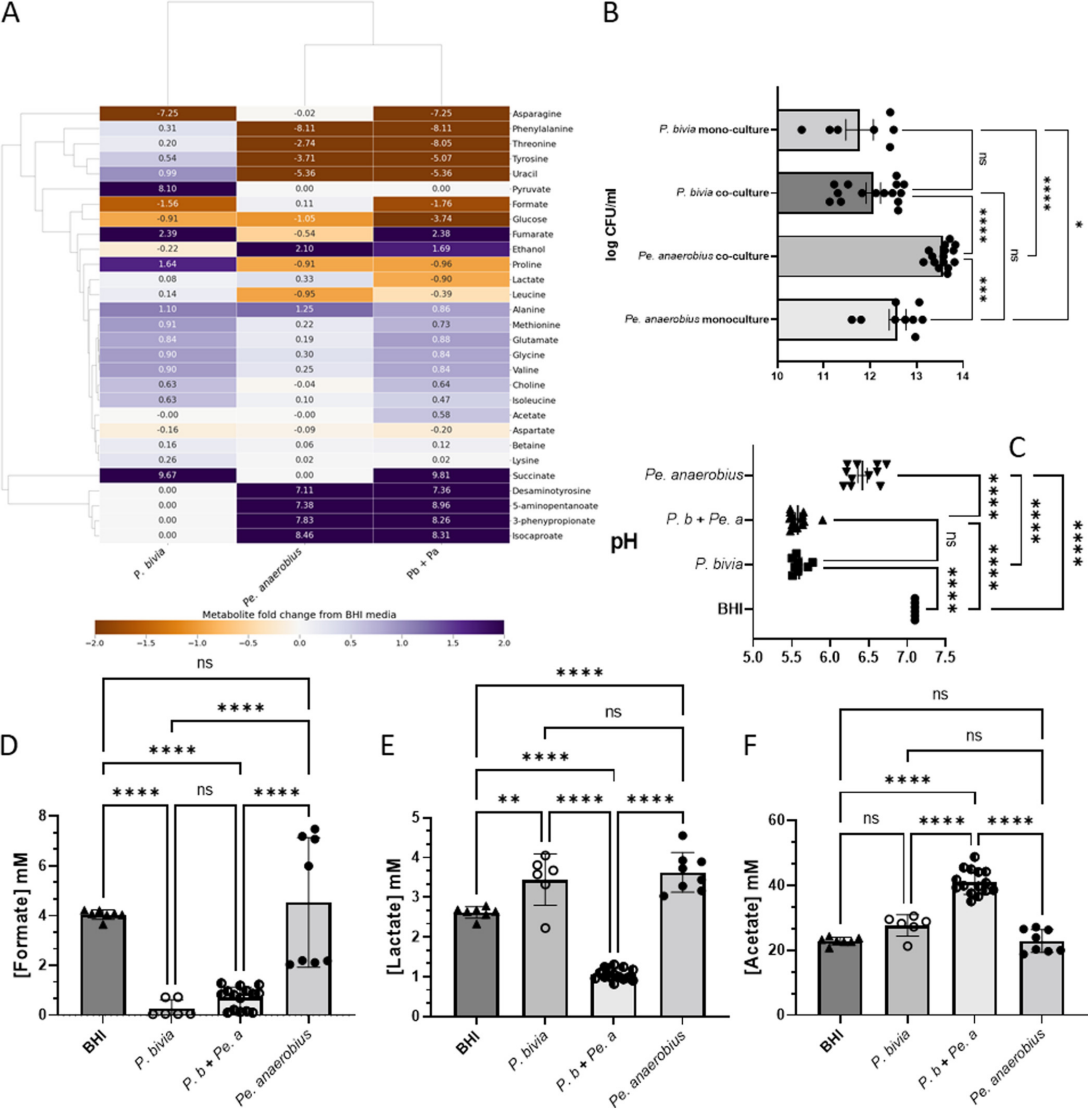
Commensal symbiosis of *Pr. bivia* and *Pe. anaerobius* in BHI generates a distinct chemical environment. (A) The heatmap compares the metabolite fold change, relative to fresh BHI plus 5% horse serum, from ^1^H NMR of spent culture media and enables the major metabolites produced by each isolate and the key differences in metabolic strategy to be revealed. (B) Coculture increases growth of *Pe. anaerobius* but not *Pr. bivia*. (C) The pH of the three spent cultures is compared with fresh BHI. Levels of formate (D), lactate (E), and acetate (F) in spent culture are shown relative to fresh BHI. Further metabolites are shown in Fig. S4 and S5. Comparisons are shown between all conditions, as determined by one-way ANOVA with Tukey correction for multiple comparisons. *, *P < *0.05; **, *P < *0.01; ***, *P < *0.001; ****, *P < *0.0001.

^1^H NMR of the spent culture from *Pr. bivia*, *Pe. anaerobius*, and a 1:1 coculture reveals that combining the two species leads to a substantial adjustment in the levels of metabolites that have previously been associated with PTB and/or shown utility in predicting patient outcomes. Both species proliferate when cultured together, although only *Pe. anaerobius* grows better than when cultured alone (Fig. 5B). This is supported by clear evidence from production and consumption of species-specific metabolites in the spent media (Fig. 5A and Fig. S4). In monoculture, only *P. bivia* consumes asparagine and produces fumarate and succinate, and this is observed in coculture, although succinate production is slightly reduced (*P = *0.0013) (Fig. 5A and Fig. S4A to C). Similarly, *Pe. anaerobius* is known to have a characteristic organic acid production profile (41), and in monoculture, of the two species, only *Pe. anaerobius* produces ethanol, 4-methylpentanoate (isocaproate), 3-(4-hydroxyphenyl)propanoate (desaminotyrosine/phloretic acid), 3-phenylpropionate (hydrocinnamate), and 5-aminopentanoate (aminovalerate) (Fig. 1A and Fig. S4D to H) and consumes threonine, tyrosine, proline, uracil, phenylalanine, and leucine (Fig. 1A and Fig. S2). This is also observed in coculture (Fig. 5A and Fig. S4D to N), with increased production also observed for 5-aminopentanoate, 3-phenylpropionate, and desaminotyrosine (Fig. 5A and Fig. S2F to H).

10.1128/msphere.00166-22.4 FIG S4 Univariate analysis of spent BHI metabolite concentrations for *Pr. bivia*/*Pe. anaerobius* coculture. Comparisons are shown between all conditions, as determined by one-way ANOVA with Tukey correction for multiple comparisons. *, *P < *0.05; **, *P < *0.01; ***, *P < *0.001; ****, *P < *0.0001. Download FIG S4, PDF file, 0.3 MB. Copyright © 2022 Horrocks et al. 2022 Horrocks et al. https://creativecommons.org/licenses/by/4.0/ This content is distributed under the terms of the Creative Commons Attribution 4.0 International license.

10.1128/msphere.00166-22.5 FIG S5 Further univariate analysis of spent BHI metabolite concentrations or culture optical density for *Pr. bivia*/*Pe. anaerobius* mono- and coculture. Comparisons are shown between all conditions, as determined by one-way ANOVA with Tukey correction for multiple comparisons. *, *P < *0.05; **, *P < *0.01; ***, *P < *0.001; ****, *P < *0.0001. Download FIG S5, PDF file, 0.2 MB. Copyright © 2022 Horrocks et al. 2022 Horrocks et al. https://creativecommons.org/licenses/by/4.0/ This content is distributed under the terms of the Creative Commons Attribution 4.0 International license.

Previously, a commensal symbiosis between *Pr. bivia* and *Pe. anaerobius* was demonstrated and ascribed to use of amino acids by *Pe. anaerobius* that were secreted by *Pr. bivia* (24). Here, ^1^H NMR identifies enrichment of BHI medium with leucine, lysine, threonine, tyrosine, phenylalanine, proline, methionine, alanine, glutamate, glycine, isoleucine, valine, choline, and uracil (Fig. 5 and Fig. S4 and S5). Of these, *Pe. anaerobius* avidly consumes threonine, tyrosine, phenylalanine, proline, and uracil and some leucine (Fig. 5 and Fig. S4). Levels of methionine, lysine, alanine, glutamate, glycine, isoleucine, and valine are also lower in the coculture spent media than for *Pr. bivia*, but since these are available in BHI normally and are not consumed in *Pe. anaerobius* monoculture, it is assumed that this reduction can also be accounted for by lower overall growth and/or altered metabolism of *P. bivia* in the combination relative to monoculture (Fig. S4 and Fig. S5).

While the benefits of coculture to *Pe. anaerobius* appear manifold and its growth in coculture is enhanced over that observed in monoculture, the reverse is not true for *Pr. bivia* (Fig. 5B), and ^1^H NMR does not detect any metabolites produced by *Pe. anaerobius* that are consumed by *P. bivia*. This supports the previous finding of a commensal relationship between the two organisms (24). There is one possible caveat to this in that, while no effect of *Pe. anaerobius* conditioned medium on *Pr. bivia* growth was observed previously (24), here we find that *Pr. bivia* metabolism is likely altered by coculture with *Pe. anaerobius*. First, while production of *Pe. anaerobius*-specific metabolites is increased in coculture relative to monoculture, the same is not true for *Pr. bivia*, with less succinate, glutamate, glycine, isoleucine, and valine than might be expected. Second, while consumption of formate by *Pr. bivia* is observed in both monoculture and coculture (Fig. 5D), lactate, produced by both species in monoculture, is found to be depleted in the coculture spent media relative to fresh BHI (Fig. 5E). Both formate and lactate are potential electron donors for anaerobic respiration, and the NMR analysis provides evidence for an increase in lactate consumption by *Pr. bivia* when *Pe. anaerobius* is present. In contrast, under the same conditions acetate production is stimulated (Fig. 5F).

The spent culture medium pH will be affected by the production/consumption of a range of organic and amino acids. Although acidification of spent culture will be limited, the relatively low levels of glucose interactions between these two species will affect the acidity of the environment (Fig. 5C). Despite production of acetate (pK_a_ 4.76) and lactate (pK_a_ 3.86), acidification by *Pe. anaerobius* is relatively modest, with a reduction by only 0.68 pH units. In contrast, both the spent *Pr. bivia* monoculture and coculture are reduced by more than one pH unit (1.51 and 1.54, respectively). In both cases substantial amounts of succinate (pK_a_ 4.2 and 5.6) are produced (22.7 mM versus 18.1 mM for monoculture versus coculture). More acetate is produced in the coculture, but there is no net lactate production. These effects combine to ensure that the spent coculture and *Pr. bivia* monoculture pH are substantially lower than that corresponding to *Pe. anaerobius*.

### Symbiosis between *Pr. bivia* and *G. vaginalis* is strain and metabolic strategy dependent.

**FIG 6:**
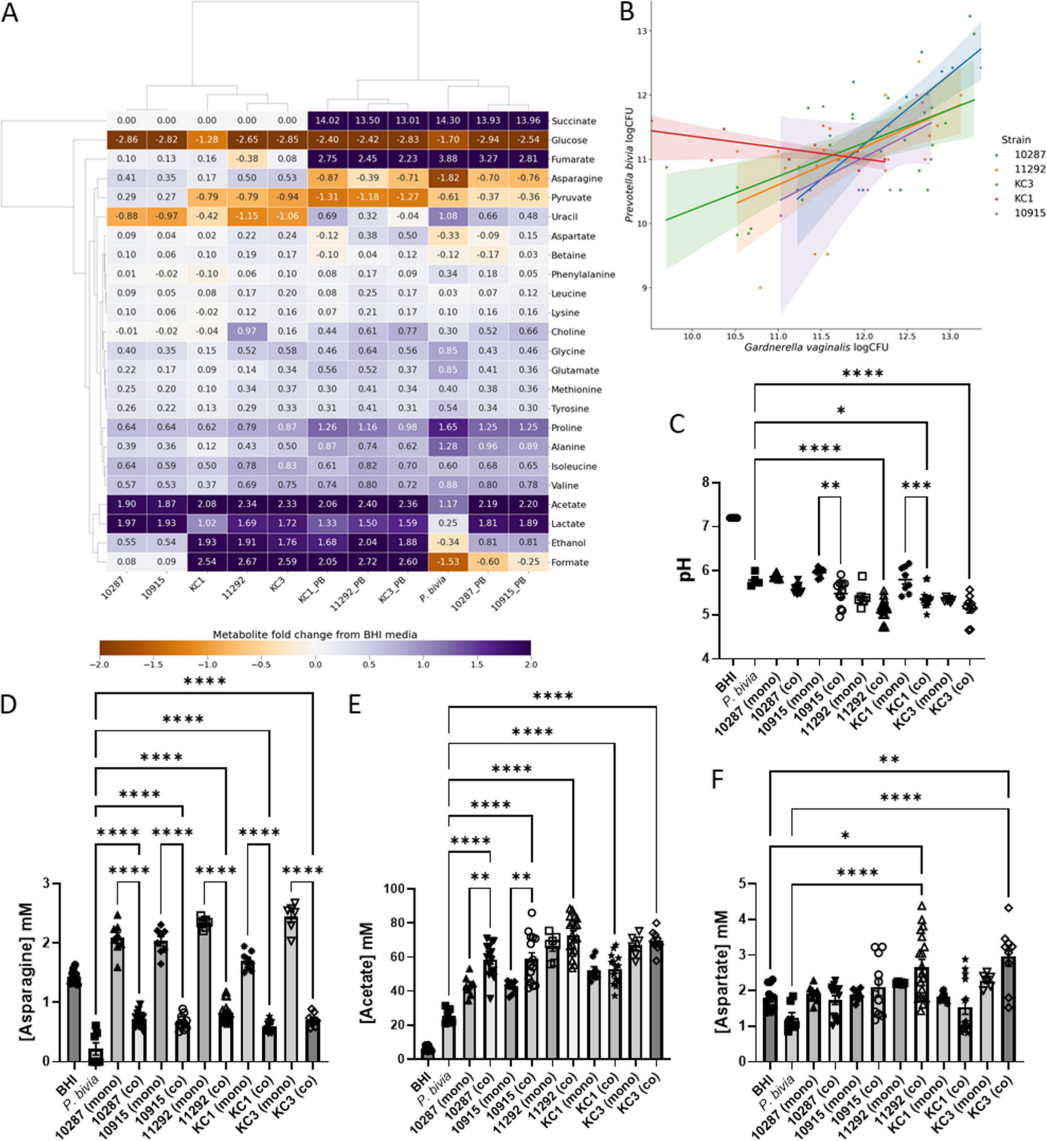
Coculture of Prevotella bivia NCTC 11156 and a panel of Gardnerella vaginalis isolates. (A) A heatmap shows the relationship between metabolite concentration fold changes, relative to fresh BHI plus 5% horse serum, detected by ^1^H NMR of spent cultures. (B) The correlation between CFU number for *Pr. bivia* and each *G. vaginalis* partner is shown for five cocultures; Spearman and Pearson *r* values are provided in Table 1. (C) Spent culture pH for mono- and cocultures as well as fresh BHI. (D) *G. vaginalis* supplies *Pr. bivia* with asparagine. (E) Acetate levels increase when bifid shunt *G. vaginalis* strains (10287 and 10915) are cocultured with *Pr. bivia*. (F) Symbiosis between *Pr. bivia* and MAF *G. vaginalis* strains produces aspartate. Comparisons are shown between each coculture and the corresponding monococultures (C to E) and also fresh BHI (F) as determined by one-way ANOVA with Tukey correction for multiple comparisons. Only *P *values <0.05 are shown. *, *P < *0.05; **, *P < *0.01; ***, *P < *0.001; ****, *P < *0.0001. Comparisons for further metabolites are shown in Fig. S6.

Five *G. vaginalis* strains (10287, 10915, 11292, KC1, and KC3), representing both BS and MAF strategies, were selected for coculture experiments with *Pr. bivia* NCTC 11156. With the exception of KC1, the only strain in the present study not of sequence variant G2 (9), positive correlations were detected between the number of CFU identified for either species when plated following coculture in BHI (Fig. 6B, Table 2), with the strongest positive relationship found for *G. vaginalis* 10287, one of the BS strains.

**TABLE 2:** *Pr. bivia* versus *G. vaginalis* coculture correlation^*a*^

*G. vaginalis* strain	No. of XY pairs	Spearman		Pearson	
		*r*	*P* (two-tailed)	*r*	*P* (two-tailed)
NCTC 10287	16	0.7917	0.0005	0.7781	0.0004
NCTC 10915	9	0.6442	0.0694	0.5419	0.1318
NCTC 11292	22	0.5457	0.0086	0.5133	0.0146
KC1	10	−0.4768	0.1645	−0.6896	0.0274
KC3	19	0.5947	0.0072	0.6392	0.0032

a Relationship between CFU counts for each species as a function of *G. vaginalis* isolate as determined by parametric Pearson or nonparametric Spearman correlation coefficients. Only for KC1 is a negative correlation between the two species found, while positive correlations exist for the remaining four isolates.

The Spearman and Pearson *r* for KC1 are both negative, indicating that when *G. vaginalis* KC1 grew well *Pr. bivia* did not, and vice versa. This is manifested in the metabolomics analysis where levels of some metabolites, known to be produced by *Pr. bivia*, notably succinate, fumarate, proline, uracil, and alanine, are highly variable (Fig. S6D to F, J, and K). There is some explanation for this phenomenon in the metabolomics data (Fig. 6A). Notably, KC1 may be the only one of the five *G. vaginalis* strains that is not capable of adequately supplying asparagine to *Pr. bivia* (Fig. 6D). As noted above, *Pr. bivia* avidly consumes asparagine, since this can be used to produce aspartate and, in turn, fumarate, which is an important electron acceptor in anaerobic respiration. Asparagine is produced in substantial amounts by all *G. vaginalis* isolates (*P < *0.0001), with the exception of KC1 (*P = *0.0333).

This is modest compared with MAF strains 11292 and KC3, which increase the availability of asparagine by 63% and 70%, respectively, such that approximately double the amount of asparagine that is consumed by *Pr. bivia* in monoculture is available in coculture. In contrast, KC1 increases the amount available by only 18.5%.

While supply of asparagine from *G. vaginalis* to *Pr. bivia* is observed for both MAF and BS strains, a further means by which BS strains, but not MAF strains, may supply *Pr. bivia* is also apparent. Unlike the BS *G. vaginalis* strains, *Pr. bivia* and all three MAF *G. vaginalis* strains consume pyruvate from BHI (Fig. 6A and Fig. S6C). With two species growing together, the metabolite data for coculture have greater variance, but considering just the data from monocultures (as described above) indicates that some pyruvate is likely secreted from 10287 (*P = *0.0035) and 10915 (*P = *0.0046). As such the BS strains differ from the MAF strains in that they avoid competition with *Pr. bivia* for pyruvate and likely supply it in coculture.

As noted above, in monoculture the MAF strains 11292 and KC3 (*P < *0.0001) and KC1 (*P < *0.05) produce more acetate than the BS strains 10287 and 10915 but less lactate. In coculture, however, acetate produced by *Pr. bivia*/10287 and *Pr. bivia*/10915 increases by 42 to 45% over that produced by *G. vaginalis* alone, while the corresponding figure for the MAF strains is between 2 and 11%. Lactate production is largely unchanged in coculture for any of the strains. Coculture with *Pr. bivia* therefore has the potential to substantially increase overall acetate levels and change the acetate/lactate ratio when BS strains are present but not MAF strains. Further, while *Pr. bivia* was confirmed to consume formate, ethanol, and aspartate by spiking experiments (Fig. S7), there is insufficient evidence here that production of these metabolites by MAF *G. vaginalis* provides substantial benefit for *Pr. bivia* with no apparent consumption of these metabolites in the respective cocultures (Fig. 6A and Fig. S6F to H). Indeed, while both 11292 (*P = *0.015) and KC3 (*P = *0.008) produce aspartate in monoculture, the amount found in the spent coculture medium is increased 2- and 3-fold, respectively (Fig. 6F). Previous work has indicated *Pr. bivia* supplies ammonia to *G. vaginalis* (25), and this suggests that MAF *G. vaginalis* performs a detoxification role by consuming both ammonia and fumarate (Fig. 6A and Fig. S6E), secreted by *Pr. bivia*, to produce aspartate (42).

10.1128/msphere.00166-22.7 FIG S7 Univariate analysis of spent BHI metabolite concentrations for *Pr. bivia* after spiking. Comparisons are shown between fresh and spent for the spiked metabolite in each case (A, C, and E), as determined by a *t* test, and between all other conditions (B, D, and F), as determined by one-way ANOVA with Tukey correction for multiple comparisons. Only *P* values <0.05 are shown. *, *P < *0.05; **, *P < *0.01; ***, *P < *0.001; ****, *P < *0.0001. Download FIG S7, PDF file, 0.06 MB. Copyright © 2022 Horrocks et al. 2022 Horrocks et al. https://creativecommons.org/licenses/by/4.0/ This content is distributed under the terms of the Creative Commons Attribution 4.0 International license.

**FIG 7:**
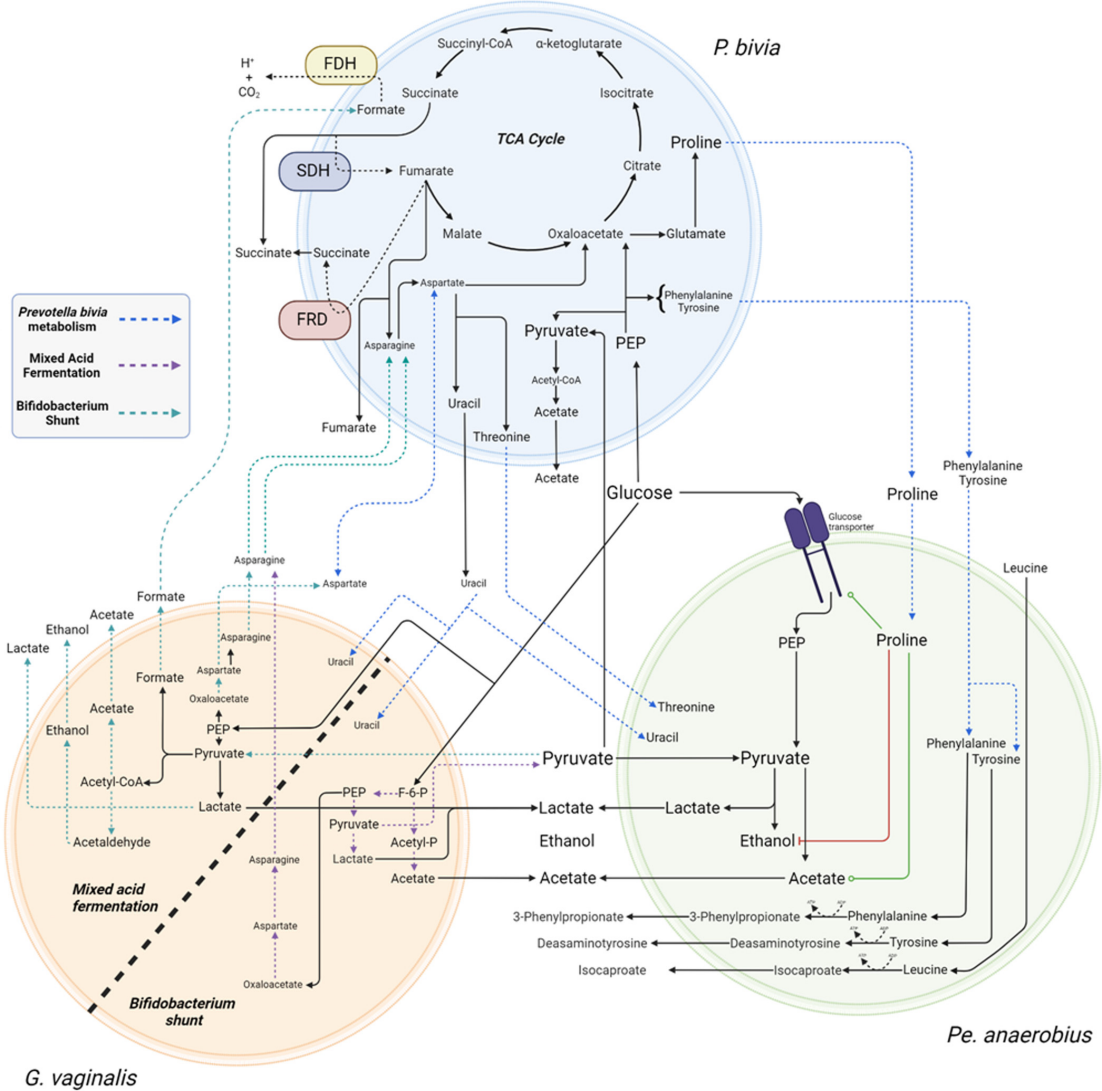
Commensal relationship of Prevotella bivia NCTC 11156 with *Pe. anaerobius* NCTC 11460 and mutualistic relationships with BS and MAF Gardnerella vaginalis. *Pr. bivia* supplies uracil, tyrosine, phenylalanine, and proline to *Pe. anaerobius*. These all stimulate glucose uptake by *Pe. anaerobius*, and increased proline availability also causes a switch from ethanol to acetate production, accounting for a 56% increase in acetate secretion. The relationship between *G. vaginalis* and *Pr. bivia* is mutualistic, with the former supplying asparagine and the latter again supplying uracil. However, the relationship between MAF or BS *G. vaginalis* strains and *Pr. bivia* will differ, with MAF strains competing with *Pr. bivia* for pyruvate but potentially supplying formate as an electron donor for anaerobic respiration. The origin of the increased aspartate found in MAF *G. vaginalis* and *Pr. bivia* coculture is as yet unclear. FDH, formate dehydrogenase; SDH, succinate dehydrogenase; FRD, fumarate reductase. Image created with BioRender.com.

While the symbiotic relationship between *Pr. bivia* and *Pe. anaerobius* is commensal in BHI, we suggest here that the relationship between *Pr. bivia* and *G. vaginalis* is mutualistic, since, as for the presumed supply of ammonia and fumarate, we show *Pr. bivia* also likely supplies *G. vaginalis* with uracil (Fig. 6 and 7 and Fig. S6J). As noted above, in monoculture *Pr. bivia* produces uracil and all five *G. vaginalis* strains consume it. Not all uracil is consumed, however, and in coculture the overall levels remaining in spent culture are intermediate between that obtained from *Pr. bivia* monoculture and that available in fresh BHI. Nevertheless, pending further investigation, there is no reason to assume uracil liberated by *Pr. bivia* is not then available to *G. vaginalis*.

The levels of other metabolites vary little between the spent monoculture and cocultures, although choline, produced by *Pr. bivia* but not *G. vaginalis* in monoculture, is further increased in three out of the five spent cocultures, 10915, 11292, and KC3 (Fig. 6A and Fig. S6N).

## DISCUSSION

The present study describes the metabolic strategies and quantifies the relative metabolites produced and consumed in BHI by both a panel of lactobacilli and a range of BV- and/or PTB-associated bacteria. Further, we characterize the effect on metabolite consumption and excretion and consequently the likely vaginal chemical environment of commensal symbiosis between *Pr. bivia* and *Pe. anaerobius* and a mutualistic symbiosis between *Pr. bivia* and *G. vaginalis*, providing mechanistic details for both. We demonstrate substantial differences in metabolite consumption/production between different strains of *G. vaginalis* that adopt either BS alone or MAF strategies and how this affects outcome of the mutualistic symbiosis with *Pr. bivia*. Below we consider the effects of the two symbiotic relationships before assessing how variation in metabolic strategy in lactobacilli, BV/PTB-associated bacteria, and symbiosis affects the vaginal chemical environment and how this may have functional impact and modify metabolite-based approaches to PTB risk prediction.

### Commensal supply of proline by *Pr. bivia* increases acetogenesis by *Pe. anaerobius*.

Commensal symbiosis of *Pr. bivia* and *Pe. anaerobius* is known to depend on provision of amino acids from the former to the latter (24). Here, we show that, in addition, uracil supply is substantial and that these amino acids are limited to threonine, tyrosine, phenylalanine, and proline, with each of these consumed avidly by *Pe. anaerobius.* All four of these amino acids have been shown to stimulate glucose uptake, with leucine and tyrosine having the greatest effect (43). Quantitative analysis of the metabolism of tyrosine, phenylalanine, and proline and its impact may help to explain the relative importance of their respective availabilities. The increased availability of tyrosine and phenylalanine is associated with a 29% increase in desaminotyrosine and a 53% increase in 3-phenylpropionate production in coculture compared with *Pe. anaerobius* conditioned medium. In contrast, proline availability increases by 595% in *Pr. bivia* conditioned medium, and this leads to a 386% increase in 5-aminopentanoate production in coculture. Proline has been shown to be capable of initiating glucose uptake, and high proline levels are associated with a switch from ethanol to acetate production, a process that generates additional ATP (43). Here, the concentration of acetate in coculture increases by 18.2 mM over the *Pe. anaerobius* monoculture, but no increase is observed for ethanol. As such, coexistence of *Pr. bivia* with *Pe. anaerobius* and/or greater availability of proline from other sources can be expected to substantially increase production of acetate (Fig. 7).

### Diversity in *G. vaginalis* metabolism influences symbiosis with *Pr. bivia*.

When originally described, *G. vaginalis* was proposed to be the sole etiological agent of BV, since it was found in 127 out of 138 cases but in none of 78 healthy women examined (27, 44). Since then more doubt has been expressed that *G. vaginalis* alone is the causative agent of BV, as its distribution is more widespread and is frequently found colonizing the vagina of healthy or nonsymptomatic women. At the same time, there is recognition that there is considerable diversity in the *G. vaginalis* genus, with both different species and clades or subgroups being proposed (29, 30, 45). The functional relevance of diversity in *G. vaginalis* has been highlighted by the finding in one study that an association between *G. vaginalis* and PTB was driven exclusively by G2 sequence variants with an association absent for other variants and the association for the genus lost when G2 variants were excluded (9). The implication from this is that associations and mechanistic links between *G. vaginalis* and PTB, if they exist, will be obscured if diversity is not considered.

It has been shown recently that *G. vaginalis* enhances the invasive potential of *Pr. bivia* (26), aiding its ascension into the uterus. A commensal metabolic symbiotic relationship between these two species was proposed over 20 years ago (25). Here, we use NMR metabolomics to characterize the symbiosis between *Pr. bivia* and *G. vaginalis*. Of the five *G. vaginalis* isolates tested here, those four that are identified as G2 sequence variants (9) benefit from a relationship that this is mutualistic rather than commensal and further show that the outcome is specific to the metabolic strategy of specific *G. vaginalis* isolates. As such, we show that diversity in *G. vaginalis* metabolism is manifested both in monoculture and coculture and has potential to alter the vaginal chemical environment. Lower lactate and higher acetate levels in the vagina are considered hallmarks of BV and are associated with sPTB (15, 17, 22). Such conditions would be consistent with a depletion of lactobacilli and increase in *G. vaginalis*, but this relative difference also describes the relationship between BS and MAF strains of *G. vaginalis*, albeit not to the same magnitude. Further, since coculture of MAF strains, but not BS strains, with *Pr. bivia* leads to an increase in aspartate production, this diversity impacts an important metabolite predictor of sPTB (15).

### Functional impact and implications for risk prediction of BV-associated bacteria and lactobacillus metabolism.

Low vaginal pH and high lactate are both associated with protective benefits, while short-chain fatty acids (SCFAs), including acetate, butyrate, and succinate (and propionate where present), have pleiotropic effects in inflammation (18–21). A recent comparison of the effects of treating cervicovaginal epithelial cells with mixtures of organic acids representing optimal (33 mM lactic acid/lactate, 4 mM acetic acid, 1 mM succinic, butyric, and propionic acids) and nonoptimal (6 mM lactate, 100 mM acetate, 20 mM succinate, and 4 mM butyrate and propionate) vaginal microbiota at pH 3.9 and pH 7 revealed that the mixture chosen to mimic BV increased basal and toll-like receptor (TLR)-induced production of proinflammatory cytokines, including tumor necrosis factor-α (TNF-α), but decreased basal production of CCL5 and IP-10 chemokines (20). When tested alone, 100 mM acetate at pH 7 largely recapitulated the effects of the BV mixture. Since the pK_a_ of acetic acid is 4.75 and those of succinic acid are 4.2 and 5.6, these will exist as the carboxylate or dicarboxylate anions at such an extreme as pH 7. As both the relative concentrations and the ionization state of the organic acids are changing under these experimental conditions, it is unclear as to the relative importance of these two factors, and the impact of acetic acid/acetate may depend on the vaginal pH, driven by relative concentrations of primarily lactic acid. The absolute and relative proportions of these two organic acids may therefore have substantial impact on the vaginal inflammatory state and need to be considered.

The description of metabolism, in pairings of *Pr. bivia* with diverse *G. vaginalis* isolates, reveals symbiosis has the potential to substantially increase the amount of acetate excreted by BS but not MAF strains. Similarly, coculture between *Pr. bivia* and *Pe. anaerobius* modulates pH, eliminates net lactate production, and increases acetate production. Together, these observations raise the prospect that coexistence of *Pr. bivia* with either of the two species affects their physiological impact.

Further, while this study is predominantly focused on the metabolism of PTB- and/or BV-associated bacteria, it is also important to consider the metabolism of lactobacilli that often dominate the vaginal microbiome, and hence contribute to the metabolite background, and their known relationships with BV/PTB-associated bacteria. Patterns of cooccurrence between L. crispatus and *G. vaginalis* have been shown to be highly exclusive (9). In contrast, *L. iners* has been shown to coexist with *G. vaginalis* at high frequency, and its dominance has been found to be associated with preterm birth (10). Since this work confirms *L. iners* is incapable of making acetate (39, 40), all acetate detected in an *L. iners*-dominated sample will originate from other bacteria, frequently *G. vaginalis*, and the change in acetate levels may be expected to be larger in such situations than observed where other lactobacilli dominate or that are considered mixed dysbiotic. This may have implications both for inflammation and risk prediction. Indeed, acetate production by lesser producers (*A. vaginae*, *Pr. bivia*, and perhaps BS *G. vaginalis*) may be easier to detect in low-acetate background, as found in *L. iners*-dominated CST compared with other backgrounds, i.e., L. crispatus, or where other lactobacilli coexist, e.g., L. rhamnosus, and the relative change will be greater. Similarly, although less abundant, succinate is produced by 9 out of 11 lactobacillus strains tested here, with none detected for *L. iners* and L. rhamnosus 1. Again, detection of succinate produced by PTB- and/or BV-associated *Pr. bivia* and BV-associated *M. curtisii* will be easier to detect in the *L. iners* CST background than in others.

A comparison between representative isolates of *L. iners-* and L. crispatus-dominated microbiomes is therefore warranted but beyond the scope of the present study. Notably, substantial variation in metabolism was observed in the two L. crispatus isolates, notably for asparagine consumption and aspartate and acetate production, and there is a need to establish the extent to which metabolism varies across a larger panel of isolates to appreciate its possible impact.

Finally, we assess whether the current study sheds any light on the protection against PTB suggested to be provided by L. acidophilus (15). Of note, L. acidophilus does make the largest amount of lactate of all the lactobacillus isolates grown here in BHI (*P < *0.0001 for all but L. rhamnosus, *P < *0.05, and L. jensenii 2, *P = *0.0047), and it produces the spent culture with the lowest pH. Lactate production is correlated with H_2_O_2_, which would inhibit anaerobes, and bacteriocins lose activity and hydrogen peroxide becomes unstable as the pH increases. Peroxide is, however, only produced in the presence of oxygen, and L. gasseri may make more H_2_O_2_, while cervicovaginal fluid has been shown to attenuate its microbicidal activity (46, 47). As such, the extent to which higher lactate production and greater ability to acidify the environment, from certain less-dominant lactobacilli, is protective against BV or PTB should be explored further, especially if able to coexist within more diverse communities.

### Conclusions.

The diversity of intraspecies BV/PTB-associated bacteria, and interspecies lactobacilli, metabolism, and commensal and mutualistic symbiotic relationships of *Pr. bivia* has the potential to alter proinflammatory acetate and other metabolites in the vaginal metabolome and consequently alter risk of bacterial vaginosis and/or spontaneous preterm birth.

## MATERIALS AND METHODS

### Isolates.

Gardnerella vaginalis 11292, 10915, and 10287, Peptostreptococcus anaerobius 11460, Prevotella bivia 11156, Atopobium vaginae 13935, and Mobiluncus curtisii 11656 were obtained from the National Collection of Type Cultures (NCTC). All other bacteria were isolated from swabs collected from pregnant women recruited with informed written consent via the INSIGHT study (NHS Human Research Authority, London City and East Research Ethics Committee 13LO/1393) or from Salisbury District Hospital (SDH) microbiology lab. Samples from SDH were received from the microbiology laboratory following diagnostic testing. All identifiers were removed by the diagnostic laboratory. The swabs were maintained at ambient temperature during transport in liquid Amies buffer and were used immediately or frozen at −80°C until use; 100 μL of the buffer solution was either plated onto tryptic soy agar (TSA) and De Man, Rogosa, and Sharpe (MRS) agar plates and incubated at 37°C for 48 h under aerobic condition or plated onto TSA, MRS agar, and Columbia blood agar (CBA), containing 5% defibrinated sheep’s blood (Oxoid), and incubated at 37°C for 48 h under anaerobic conditions as outlined below. Single colonies were streaked to purity and identified using matrix-assisted laser desorption ionization–time of flight (MALDI-TOF) spectrometry (MALDI Biotyper; Bruker Daltonics GmbH & Co.).

### Bacterial culture.

All *G. vaginalis* isolates, *Pe. anaerobius*, *Pr. bivia*, *M. curtisii*, and *A. vaginae* were plated onto CBA (Oxoid, Hampshire, UK) containing 5% defibrinated sheep’s blood (Oxoid) and incubated at 37°C for 48 h under anaerobic conditions generated using Thermo Scientific Oxoid AnaeroGen. *L. iners* was plated under the same conditions for 72 h. All other *Lactobacillus* species were plated onto MRS agar (Sigma-Aldrich) and incubated at 37°C for 48 h under anaerobic conditions. For initial overnight cultures a 1-μL loop of culture was used to inoculate 5 mL of brain heart infusion (BHI) medium with 5% horse serum and incubated at 37°C for 48 h under anaerobic conditions without shaking. For monoculture samples, 50 μL of overnight culture was added to 5 mL of fresh BHI with 5% horse serum and incubated at 37°C for 48 h under anaerobic conditions without shaking. For coculture of *Pr. bivia* with *G. vaginalis* or *Pe. anaerobius*, from overnight cultures, a 1:1 mix of each species was used to inoculate 5 mL of fresh BHI with 5% horse serum and incubated at 37°C for 48 h under anaerobic conditions without shaking. CFU counts were obtained by plating serial dilutions of coculture onto CBA, and plates were incubated for 48 h for *G. vaginalis* and *Pr. bivia* or for 40 h for *Pe. anaerobius* and *Pr. bivia*. *Pr. bivia* was distinguished from either *G. vaginalis* or *Pe. anaerobius* by colony morphology. For each condition, at least eight independent replicate samples were desired, but fastidious growth led to some samples being discarded to avoid introducing undue variation into the analysis of growth strategies.

### CVF samples for NMR analysis.

Samples were obtained from *n* = 96 pregnant women (between 10 and 24 weeks gestation) as part of the INSIGHT study with written consent (15, 48). A Dacron swab was used to obtain cervicovaginal fluid (CVF) from the posterior fornix via speculum examination for approximately 10 s to achieve saturation and then inserted into 750 μL of standard phosphate-buffered saline solution containing protease inhibitors (Complete; Roche Diagnostics GmbH, Germany). This was immediately transported on ice to the laboratory. The swab was removed, placed in a clean tube, vortexed for 10 s, and centrifuged (2,600 × *g* for 10 min at 4°C). Resultant fluid was collected and added to the fluid in the original tube. This was mixed and centrifuged for a further 10 min to remove cell debris. Cell-free supernatant from CVF swabs (100 μL) was immersed in liquid nitrogen and lyophilized at −58°C overnight. The samples were resuspended in 600 μL of D_2_O plus 0.05 wt% TSPd-4. The pH of all samples was adjusted to within pH 7 ± 0.2 using NaOH or HCl as required.

### NMR metabolomics.

For preparation of samples to be used in metabolomics, bacterial cultures were pelleted by centrifuge at 5,000 rpm at 4°C. Supernatant was filtered with 0.22-μm membrane to remove any bacterial cells and large debris and was stored at −80°C until use. To aid suppression of the water signal and deuterium lock and act as an internal reference, 60 μL of D_2_O plus 3-(trimethylsilyl)propionic-2,2,3,3-d4 acid sodium salt (TSP-d4) was added to 570 μL of filtered supernatant. The pH of all samples was adjusted using NaOH to within 0.2 pH units of the BHI medium control. ^1^H NMR spectra were recorded on a Bruker 600 MHz Bruker Avance III NMR spectrometer (Bruker BioSpin, Coventry, United Kingdom) equipped with a 5-mm ^1^H, ^13^C, ^15^N TCI Prodigy Probe and a cooled sample changer with all samples kept at 4°C. The one-dimensional spectra were acquired under automation at a temperature of 298K using Carr-Purcell-Meiboom-Gill presaturation (CMPG) pulse sequence (cpmgrp1). The parameters of spectra acquisition are 32 transients, a spectral width of 20.83 ppm, and 65,536 data points. For assignment of metabolite peaks, additional spectra, total correlation spectroscopy (TOCSY), ^1^H-^13^C heteronuclear single quantum correlation spectroscopy, and J-resolved spectroscopy (JRES) were acquired from a pooled sample containing a small volume of all samples. Resonance positions are quoted in parts per million with respect to the methyl peak of TSP-d4 at 0.0 ppm.

All spectra were Fourier transformed in Bruker software and adjusted using automatic baseline correction and phasing in Bruker TopSpin 4.1.3. Multiple databases were used for the assignment of metabolites: Chenomx NMR suite software (Chenomx Inc, Canada), Human Metabolome Database (HMDB), and Biological Magnetic Resonance Data Bank (BMRB) (49). To convert NMR intensity to millimolar concentrations, the Chenomx software program was used, calibrated to the concentration of TSP-d4 present in the sample and adjusted for dilution by D_2_O.

### MIC testing.

The MICs were measured using a broth microdilution method in polypropylene plates (Greiner). From an overnight culture in BHI, 100 μL of bacterial culture totaling an optical density at 600 nm (OD_600_) of 0.1 was added to 100 μL of BHI medium, with 5% horse serum, containing antibiotic. After 48 h of incubation at 37°C under anaerobic conditions, the optical density at a wavelength of 600 nm was read. The lowest concentration of antibiotic where there was no growth (OD_600_ < 0.1) determined the MIC.

### Sequencing.

All isolates identified as *G. vaginalis* from MALDI-TOF were also confirmed through whole-genome sequencing. DNA was extracted from overnight culture in BHI using the GenElute bacterial genomic DNA kits (Sigma-Aldrich). DNA was tagged and multiplexed with the Nextera XT DNA kit (Illumina, San Diego, CA) and sequenced by Public Health England Genomic Services and Development Unit (PHE-GSDU) on an Illumina (HiSeq 2500) with paired-end read lengths of 150 bp. A minimum 150 Mb of Q30 quality data was obtained for each isolate. FastQ files were quality trimmed using Trimmomatic (50). SPAdes 3.1.1 was used to produce draft chromosomal assemblies, and contigs of less than 1 kb were filtered out (51). Whole-genome alignment and phylogenetic tree generation were performed using progressive alignment in Mauve version 20150226 build 10. Tree visualization was performed in FigTree version 1.4.3.

### Associated content.

Phylogeny analysis of the *G. vaginalis* strains included in the present study and further comparison of metabolites produced by BV associated bacteria, lactobacilli, and the effect of coculture is provided as Fig. S1 to S7 in the supplemental material.

### Data availability.

Genome sequences for *G. vaginalis* isolates KC1 to KC4 have been deposited with NCBI GenBank with accession numbers JAJNOU000000000, JAJNOT000000000, JAJNOW000000000, and JAJNOV000000000. Raw NMR data files are available from figshare at https://figshare.com/authors/Victoria_Horrocks/12033368.

## References

[1] Onderdonk AB, Delaney ML, Fichorova RN. 2016. The human microbiome during bacterial vaginosis. Clin Microbiol Rev 29:223–238. doi:10.1128/CMR.00075-15. 26864580 PMC4786887

[2] Guaschino S, De Seta F, Piccoli M, Maso G, Alberico S. 2006. Aetiology of preterm labour: bacterial vaginosis. BJOG 113:46–51. doi:10.1111/j.1471-0528.2006.01122.x. 17206964

[3] Leitich H, Kiss H. 2007. Asymptomatic bacterial vaginosis, and intermediate flora as risk factors for adverse pregnancy outcome. Best Pract Res Clin Obstet Gynaecol 21:375–390. doi:10.1016/j.bpobgyn.2006.12.005. 17241817

[4] US Preventive Services Task Force. 2020. Screening for bacterial vaginosis in pregnant persons to prevent preterm delivery. JAMA 323:1286–1292. doi:10.1001/jama.2020.2684. 32259236

[5] Ravel J, Gajer P, Abdo Z, Schneider GM, Koenig SSK, McCulle SL, Karlebach S, Gorle R, Russell J, Tacket CO, Brotman RM, Davis CC, Ault K, Peralta L, Forney LJ. 2011. Vaginal microbiome of reproductive-age women. Proc Natl Acad Sci USA 108:4680–4687. doi:10.1073/pnas.1002611107. 20534435 PMC3063603

[6] Romero R, Hassan SS, Gajer P, Tarca AL, Fadrosh DW, Bieda J, Chaemsaithong P, Miranda J, Chaiworapongsa T, Ravel J. 2014. The vaginal microbiota of pregnant women who subsequently have spontaneous preterm labor and delivery and those with a normal delivery at term. Microbiome 2:18. doi:10.1186/2049-2618-2-18. 24987521 PMC4066267

[7] DiGiulio DB, Callahan BJ, McMurdie PJ, Costello EK, Lyell DJ, Robaczewska A, Sun CL, Goltsman DSA, Wong RJ, Shaw G, Stevenson DK, Holmes SP, Relman DA. 2015. Temporal and spatial variation of the human microbiota during pregnancy. Proc Natl Acad Sci USA 112:11060–11065. doi:10.1073/pnas.1502875112. 26283357 PMC4568272

[8] MacIntyre DA, et al. 2015. The vaginal microbiome during pregnancy and the postpartum period in a European population. Sci Rep 5:1–9. 10.1038/srep08988 PMC4355684 25758319

[9] Callahan BJ, DiGiulio DB, Goltsman DSA, Sun CL, Costello EK, Jeganathan P, Biggio JR, Wong RJ, Druzin ML, Shaw GM, Stevenson DK, Holmes SP, Relman DA. 2017. Replication and refinement of a vaginal microbial signature of preterm birth in two racially distinct cohorts of US women. Proc Natl Acad Sci USA 114:9966–9971. doi:10.1073/pnas.1705899114. 28847941 PMC5604014

[10] Kindinger LM, Bennett PR, Lee YS, Marchesi JR, Smith A, Cacciatore S, Holmes E, Nicholson JK, Teoh TG, MacIntyre DA. 2017. The interaction between vaginal microbiota, cervical length, and vaginal progesterone treatment for preterm birth risk. Microbiome 5:6. doi:10.1186/s40168-016-0223-9. 28103952 PMC5244550

[11] Stafford GP, et al. 2017. Spontaneous preterm birth is associated with differential expression of vaginal metabolites by lactobacilli dominated microflora. Front Physiol 8:615. doi:10.3389/fphys.2017.00615. 28878691 PMC5572350

[12] Elovitz MA, Gajer P, Riis V, Brown AG, Humphrys MS, Holm JB, Ravel J. 2019. Cervicovaginal microbiota and local immune response modulate the risk of spontaneous preterm delivery. Nat Commun 10:1305. doi:10.1038/s41467-019-09285-9. 30899005 PMC6428888

[13] Fettweis JM, Serrano MG, Brooks JP, Edwards DJ, Girerd PH, Parikh HI, Huang B, Arodz TJ, Edupuganti L, Glascock AL, Xu J, Jimenez NR, Vivadelli SC, Fong SS, Sheth NU, Jean S, Lee V, Bokhari YA, Lara AM, Mistry SD, Duckworth RA, Bradley SP, Koparde VN, Orenda XV, Milton SH, Rozycki SK, Matveyev AV, Wright ML, Huzurbazar SV, Jackson EM, Smirnova E, Korlach J, Tsai Y-C, Dickinson MR, Brooks JL, Drake JI, Chaffin DO, Sexton AL, Gravett MG, Rubens CE, Wijesooriya NR, Hendricks-Muñoz KD, Jefferson KK, Strauss JF, Buck GA. 2019. The vaginal microbiome and preterm birth. Nat Med 25:1012–1021. doi:10.1038/s41591-019-0450-2. 31142849 PMC6750801

[14] Serrano MG, Parikh HI, Brooks JP, Edwards DJ, Arodz TJ, Edupuganti L, Huang B, Girerd PH, Bokhari YA, Bradley SP, Brooks JL, Dickinson MR, Drake JI, Duckworth RA, Fong SS, Glascock AL, Jean S, Jimenez NR, Khoury J, Koparde VN, Lara AM, Lee V, Matveyev AV, Milton SH, Mistry SD, Rozycki SK, Sheth NU, Smirnova E, Vivadelli SC, Wijesooriya NR, Xu J, Xu P, Chaffin DO, Sexton AL, Gravett MG, Rubens CE, Hendricks-Muñoz KD, Jefferson KK, Strauss JF, Fettweis JM, Buck GA. 2019. Racioethnic diversity in the dynamics of the vaginal microbiome during pregnancy. Nat Med 25:1001–1011. doi:10.1038/s41591-019-0465-8. 31142850 PMC6746180

[15] Flaviani F, Hezelgrave NL, Kanno T, Prosdocimi EM, Chin-Smith E, Ridout AE, von Maydell DK, Mistry V, Wade WG, Shennan AH, Dimitrakopoulou K, Seed PT, Mason AJ, Tribe RM. 2021. Cervicovaginal microbiota and metabolome predict preterm birth risk in an ethnically diverse cohort. JCI Insight 6:e149257. doi:10.1172/jci.insight.149257. PMC8410012 34255744

[16] Pace RM, Chu DM, Prince AL, Ma J, Seferovic MD, Aagaard KM. 2021. Complex species and strain ecology of the vaginal microbiome from pregnancy to postpartum and association with preterm birth. Med (N Y) 2:1027–1049. doi:10.1016/j.medj.2021.06.001. 34617072 PMC8491999

[17] Amabebe E, Reynolds S, Stern V, Stafford G, Paley M, Anumba DOC. 2016. Cervicovaginal fluid acetate: a metabolite marker of preterm birth in symptomatic pregnant women. Front Med 3:48. doi:10.3389/fmed.2016.00048. PMC5056530 27777928

[18] Aldunate M, Srbinovski D, Hearps AC, Latham CF, Ramsland PA, Gugasyan R, Cone RA, Tachedjian G. 2015. Antimicrobial and immune modulatory effects of lactic acid and short chain fatty acids produced by vaginal microbiota associated with eubiosis and bacterial vaginosis. Front Physiol 6:164. doi:10.3389/fphys.2015.00164. 26082720 PMC4451362

[19] Mirmonsef P, Zariffard MR, Gilbert D, Makinde H, Landay AL, Spear GT. 2012. Short-chain fatty acids induce pro-inflammatory cytokine production alone and in combination with Toll-like receptor ligands. Am J Reprod Immunol 67:391–400. doi:10.1111/j.1600-0897.2011.01089.x. 22059850 PMC3288536

[20] Li M, van Esch BCAM, Wagenaar GTM, Garssen J, Folkerts G, Henricks PAJ. 2018. Pro- and anti-inflammatory effects of short chain fatty acids on immune and endothelial cells. Eur J Pharmacol 831:52–59. doi:10.1016/j.ejphar.2018.05.003. 29750914

[21] Delgado-Diaz DJ, Tyssen D, Hayward JA, Gugasyan R, Hearps AC, Tachedjian G. 2019. Distinct immune responses elicited from cervicovaginal epithelial cells by lactic acid and short chain fatty acids associated with optimal and non-optimal vaginal microbiota. Front Cell Infect Microbiol 9:446. doi:10.3389/fcimb.2019.00446. 31998660 PMC6965070

[22] Yeoman CJ, Thomas SM, Miller MEB, Ulanov AV, Torralba M, Lucas S, Gillis M, Cregger M, Gomez A, Ho M, Leigh SR, Stumpf R, Creedon DJ, Smith MA, Weisbaum JS, Nelson KE, Wilson BA, White BA. 2013. A multi-omic systems-based approach reveals metabolic markers of bacterial vaginosis and insight into the disease. PLoS One 8:e56111. doi:10.1371/journal.pone.0056111. 23405259 PMC3566083

[23] Spiegel CA, Amsel R, Eschenbach D, Schoenknecht F, Holmes KK. 1980. Anaerobic bacteria in nonspecific vaginitis. N Engl J Med 303:601–607. doi:10.1056/NEJM198009113031102. 6967562

[24] Pybus V, Onderdonk AB. 1998. A commensal symbiosis between *Prevotella bivia* and *Peptostreptococcus anaerobius* involves amino acids: potential significance to the pathogenesis of bacterial vaginosis. FEMS Immun Med Microbiol 22:317–327. doi:10.1111/j.1574-695X.1998.tb01221.x. 9879923

[25] Pybus V, Onderdonk AB. 1997. Evidence for a commensal, symbiotic relationship between *Gardnerella vaginalis* and *Prevotella bivia* involving ammonia: potential significance for bacterial vaginosis. J Infect Dis 175:406–413. doi:10.1093/infdis/175.2.406. 9203662

[26] Gilbert NM, Lewis WG, Li G, Sojka DK, Lubin JB, Lewis AL. 2019. *Gardnerella vaginalis* and *Prevotella bivia* trigger distinct and overlapping phenotypes in a mouse model of bacterial vaginosis. J Infect Dis 220:1099–1108. doi:10.1093/infdis/jiy704. 30715405 PMC6736442

[27] Morrill S, Gilbert NM, Lewis AL. 2020. *Gardnerella vaginalis* as a cause of bacterial vaginosis: appraisal of the evidence from *in vivo* models. Front Cell Infect Microbiol 10:168. doi:10.3389/fcimb.2020.00168. 32391287 PMC7193744

[28] Schmidt TSB, Matias Rodrigues JF, von Mering C. 2014. Ecological consistency of SSU rRNA-based operational taxonomic units at a global scale. PLoS Comput Biol 10:e1003594. doi:10.1371/journal.pcbi.1003594. 24763141 PMC3998914

[29] Ahmed A, Earl J, Retchless A, Hillier SL, Rabe LK, Cherpes TL, Powell E, Janto B, Eutsey R, Hiller NL, Boissy R, Dahlgren ME, Hall BG, Costerton JW, Post JC, Hu FZ, Ehrlich GD. 2012. Comparative genomic analyses of 17 clinical isolates of *Gardnerella vaginalis* provide evidence of multiple genetically isolated clades consistent with subspeciation into genovars. J Bacteriol 194:3922–3937. doi:10.1128/JB.00056-12. 22609915 PMC3416530

[30] Schellenberg JJ, Jayaprakash TP, Gamage NW, Patterson MH, Vaneechoutte M, Hill JE. 2016. *Gardnerella vaginalis* subgroups defined by cpn60 sequencing and sialidase activity in isolates from Canada, Belgium and Kenya. PLoS One 11:e0146510. doi:10.1371/journal.pone.0146510. 26751374 PMC4709144

[31] Garcia EM, Serrano MG, Edupuganti L, Edwards DJ, Buck GA, Jefferson KK. 2021. Sequence comparison of vaginolysin from different *Gardnerella* species. Pathogens 10:86. doi:10.3390/pathogens10020086. 33498226 PMC7909246

[32] Geshnizgani AM, Onderdonk AB. 1992. Defined medium simulating genital tract secretions for growth of vaginal microflora. J Clin Microbiol 30:1323–1326. doi:10.1128/jcm.30.5.1323-1326.1992. 1583140 PMC265277

[33] Marangoni A, Laghi L, Zagonari S, Patuelli G, Zhu C, Foschi C, Morselli S, Pedna MF, Sambri V. 2021. New insights into vaginal environment during pregnancy. Front Mol Biol 8:656844. doi:10.3389/fmolb.2021.656844. PMC8165225 34079816

[34] Gavini F, Van Esbroeck M, Touzel JP, Fourment A, Goossens H. 1996. Detection of fructose-6-phosphate phosphoketolase (F6PPK), a key enzyme of the bifid-shunt, in *Gardnerella vaginalis*. Anaerobe 2:191–193. doi:10.1006/anae.1996.0025.

[35] Cruden DL, Galask RP. 1988. Reduction of trimethylamine oxide to trimethylamine by Mobiluncus strains isolated from patients with bacterial vaginosis. Microbial Ecol Health Dis 1:95–100. doi:10.3109/08910608809140187.

[36] Spiegel CA, Roberts M. 1984. *Mobiluncus* gen. nov. *Mobiluncus curtisii* subsp. *curtisii* sp. nov. *Mobiluncus curtisii* subsp. *holmesii* subsp. nov., and *Mobiluncus mulieris* sp. nov., curved rods from the human vagina. Int J Syst Bacteriol 34:177–184. doi:10.1099/00207713-34-2-177.

[37] Holdeman LV, Johnson JL. 1977. *Bacteroides disiens* sp. nov. and *Bacteroides bivius* sp. nov. from human clinical infections. Int J Syst Bacteriol 27:337–345. doi:10.1099/00207713-27-4-337.

[38] Ruzal SM (ed). 2019. Lactobacillus genomics and metabolic engineering. Caister Academic Press, Norfolk, United Kingdom.

[39] Macklaim JM, Gloor GB, Anukam KC, Cribby S, Reid G. 2011. At the crossroads of vaginal health and disease, the genome sequence of *Lactobacillus iners* AB-1. Proc Natl Acad Sci USA 108:4688–4695. doi:10.1073/pnas.1000086107. 21059957 PMC3063587

[40] France MT, Mendes-Soares H, Forney LJ. 2016. Genomic comparisons of *Lactobacillus crispatus* and *Lactobacillus iners* reveal potential ecological drivers of community composition in the vagina. Appl Environ Microbiol 82:7063–7073. doi:10.1128/AEM.02385-16. 27694231 PMC5118917

[41] Turgeon DK, Bartley SL, Dowell VR. 1990. Use of modified norleucine-tyrosine broth in identification of *Peptostreptococcus anaerobius*. J Clin Microbiol 28:2120–2121. doi:10.1128/jcm.28.9.2120-2121.1990. 2229394 PMC268117

[42] Schleicher L, Herdan S, Fritz G, Trautmann A, Seifert J, Steuber J. 2021. Central carbon metabolism, sodium-motive electron transfer, and ammonium formation by the vaginal pathogen *Prevotella bivia*. Int J Mol Sci 22:11925. doi:10.3390/ijms222111925. 34769356 PMC8585091

[43] Curtis MA, Wittenberger CL, Thompson J. 1987. Proline requirement of glucose utilisation by *Peptostreptococcus anaerobius* ATCC 27337. Infect Immun 55:352–357. doi:10.1128/iai.55.2.352-357.1987. 3804441 PMC260333

[44] Gardner JL, Dukes CD. 1955. *Haemophilus vaginalis* vaginitis: a newly defined specific infection previously classified non-specific vaginitis. Am J Obstet Gynecol 69:962–976. doi:10.1016/0002-9378(55)90095-8. 14361525

[45] Vaneechoutte M, Guschin A, Van Simaey L, Gansemans Y, Van Nieuwerburgh F, Cools P. 2019. Emended description of *Gardnerella vaginalis* and description of *Gardnerella leopoldii* sp. nov., *Gardnerella piotii* sp. nov. and *Gardnerella swidsinskii* sp. nov., with delineation of 13 genomic species within the genus Gardnerella. Int J Syst Evol Microbiol 69:679–687. doi:10.1099/ijsem.0.003200. 30648938

[46] Strus M, Brzychczy-Włoch M, Gosiewski T, Kochan P, Heczko PB. 2006. The *in vitro* effect of hydrogen peroxide on vaginal microbial communities. FEMS Immunol Med Microbiol 48:56–63. doi:10.1111/j.1574-695X.2006.00120.x. 16965352

[47] O'Hanlon DE, Lanier BR, Moench TR, Cone RA. 2010. Cervicovaginal fluid and semen block the microbicidal activity of hydrogen peroxide produced by vaginal lactobacilli. BMC Infect Dis 10:120. doi:10.1186/1471-2334-10-120. 20482854 PMC2887447

[48] Hezelgrave NL, Seed PT, Chin-Smith EC, Ridout AE, Shennan AH, Tribe RM. 2020. Cervicovaginal natural antimicrobial expression in pregnancy and association with spontaneous preterm birth. Sci Rep 10:12018. doi:10.1038/s41598-020-68329-z. 32694552 PMC7374562

[49] Ulrich EL, Akutsu H, Doreleijers JF, Harano Y, Ioannidis YE, Lin J, Livny M, Mading S, Maziuk D, Miller Z, Nakatani E, Schulte CF, Tolmie DE, Kent Wenger R, Yao H, Markley JL. 2008. BioMagResBank. Nucleic Acids Res 36:D402–D408. doi:10.1093/nar/gkm957. 17984079 PMC2238925

[50] Bolger AM, Lohse M, Usadel B. 2014. Trimmomatic: a flexible trimmer for Illumina sequence data. Bioinformatics 30:2114–2120. doi:10.1093/bioinformatics/btu170. 24695404 PMC4103590

[51] Bankevich A, Nurk S, Antipov D, Gurevich AA, Dvorkin M, Kulikov AS, Lesin VM, Nikolenko SI, Pham S, Prjibelski AD, Pyshkin AV, Sirotkin AV, Vyahhi N, Tesler G, Alekseyev MA, Pevzner PA. 2012. SPAdes: a new genome assembly algorithm and its applications to single-cell sequencing. J Comput Biol 19:455–477. doi:10.1089/cmb.2012.0021. 22506599 PMC3342519

[52] O'Hanlon DE, Come RA, Moench TR. 2019. Vaginal pH measured in vivo: lactobacilli determine pH and lactic acid concentration. BMC Microbiol 19:13. doi:10.1186/s12866-019-1388-8. 30642259 PMC6332693

