# Supplementary figures and images for: Nuclear Magnetic Resonance Metabolomics of Symbioses between Bacterial Vaginosis-Associated Bacteria
Source: mSphere. 2022 May 2;7(3):e00166-22. doi: 10.1128/msphere.00166-22 (PMC9241533; doi:10.1128/msphere.00166-22)

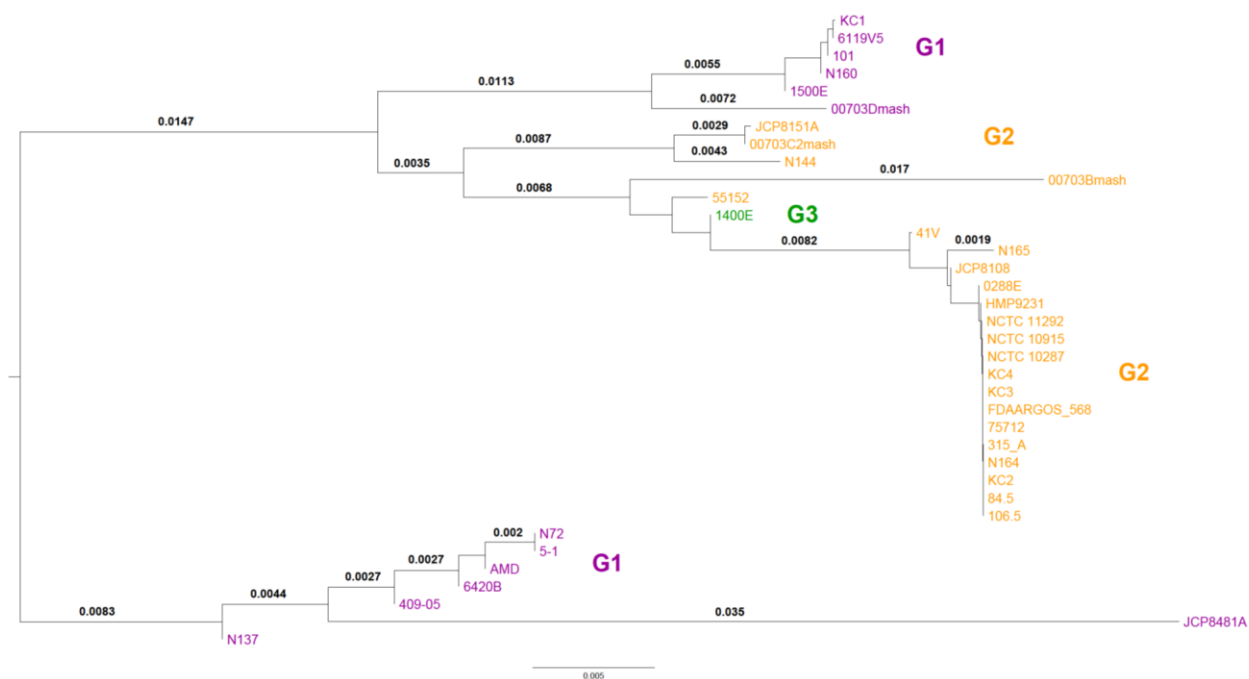

Supplement: FIG S1 [file msphere.00166-22-s0001.pdf]

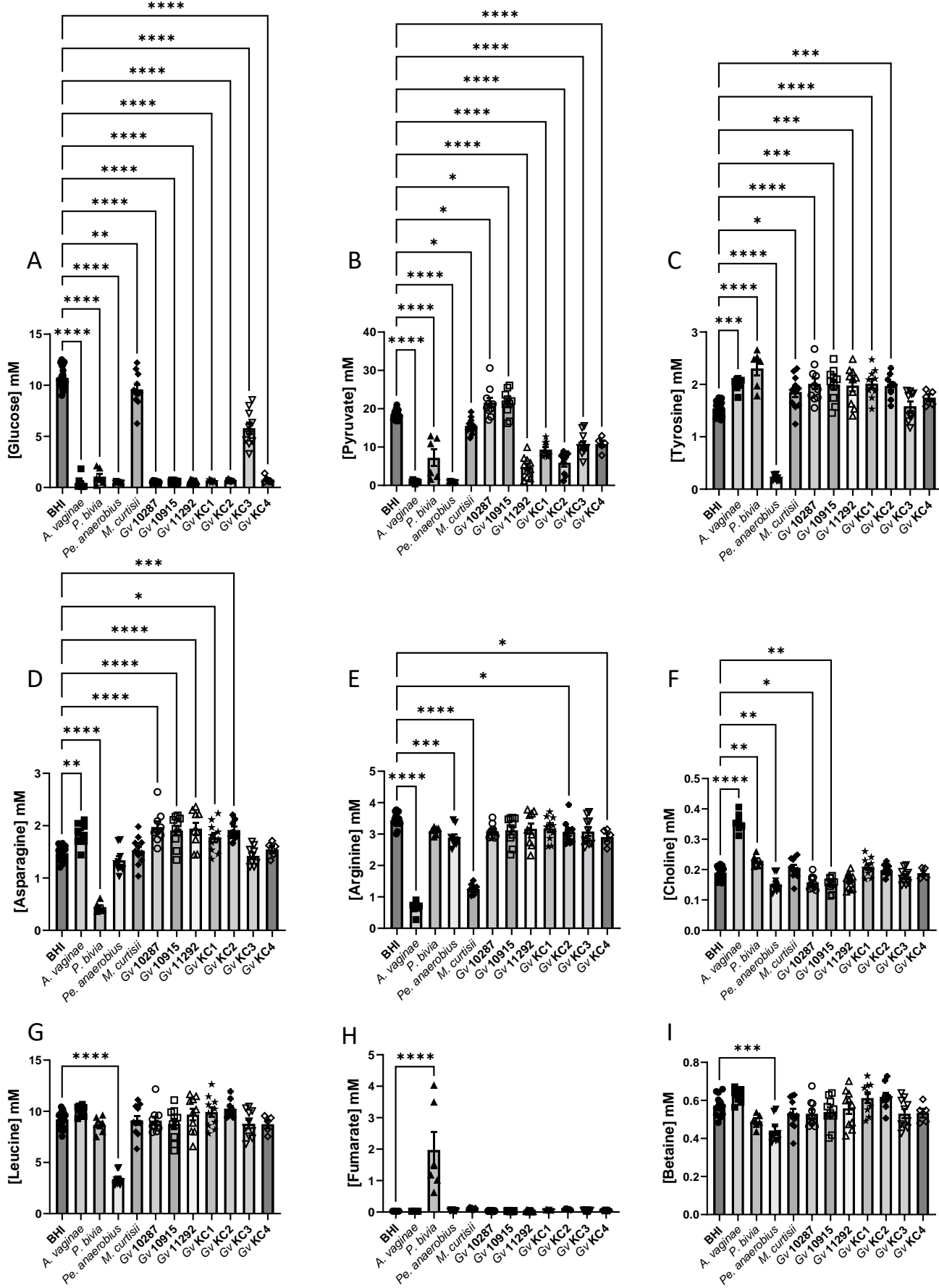

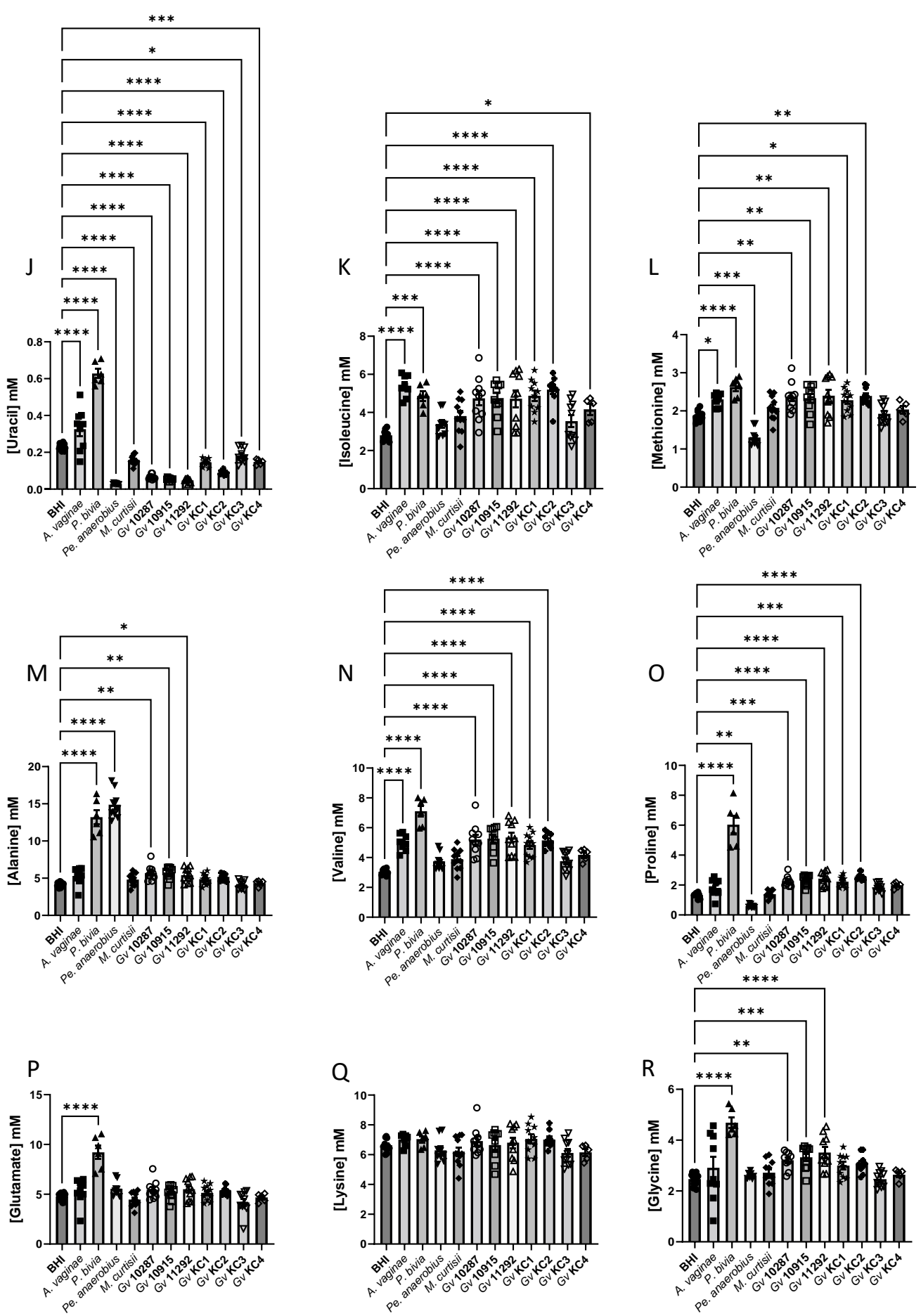

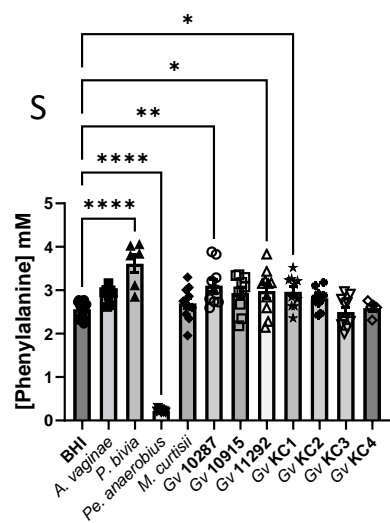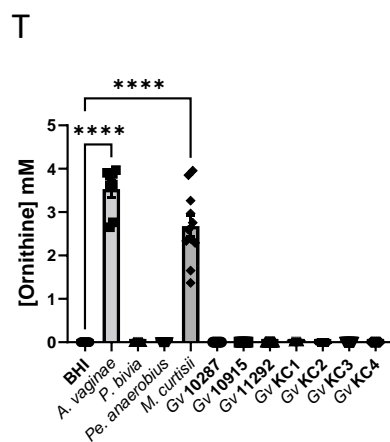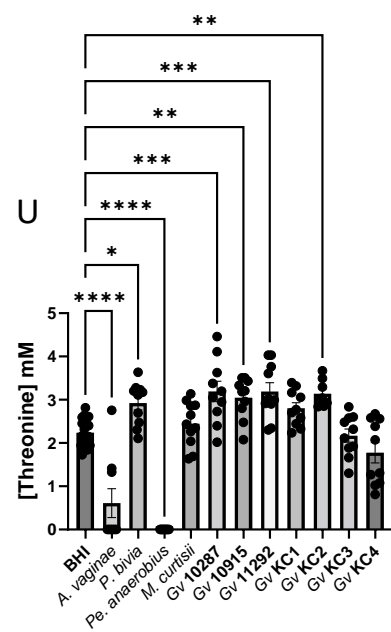

Supplement: FIG S2 [file msphere.00166-22-s0003.pdf]

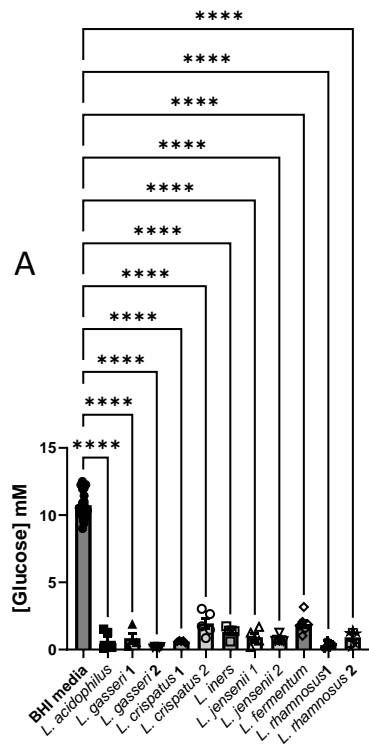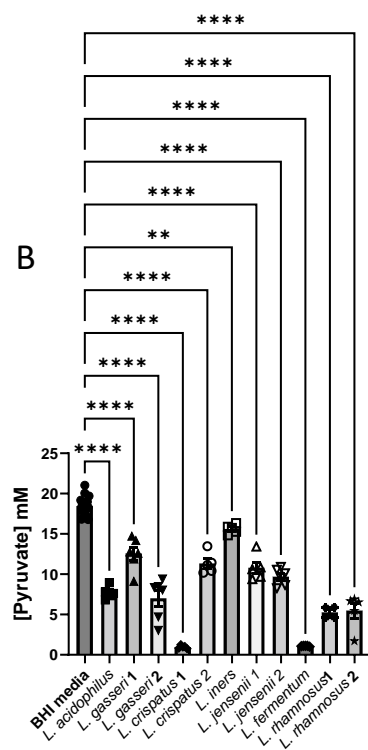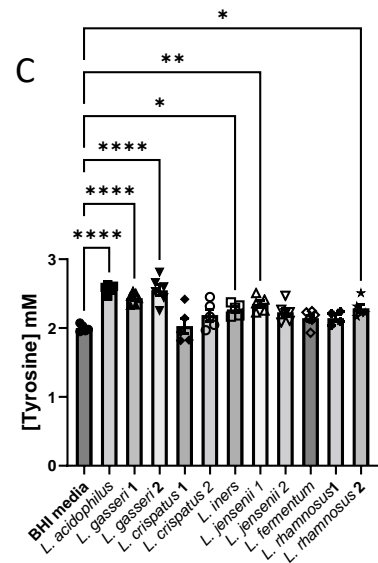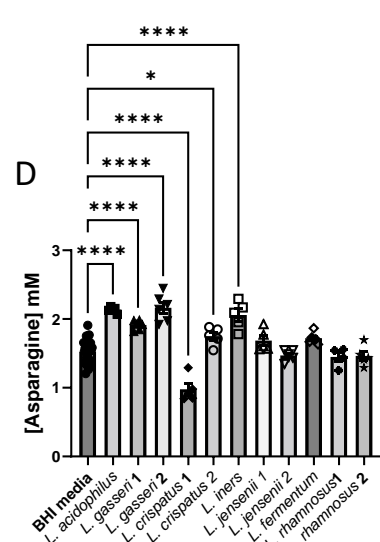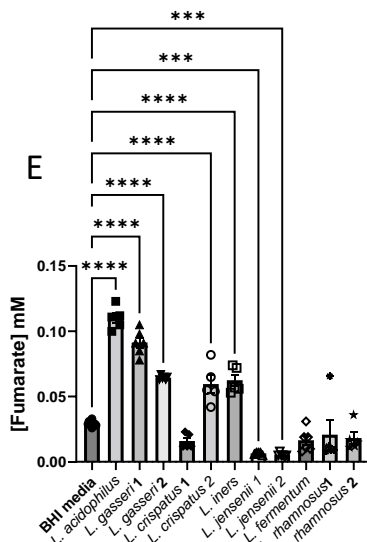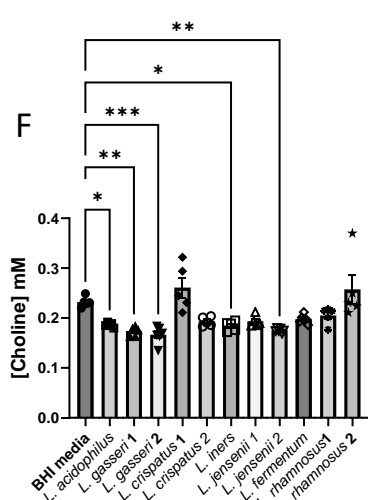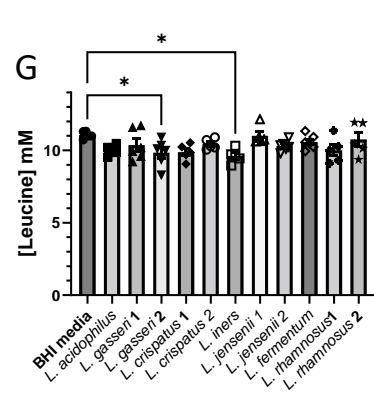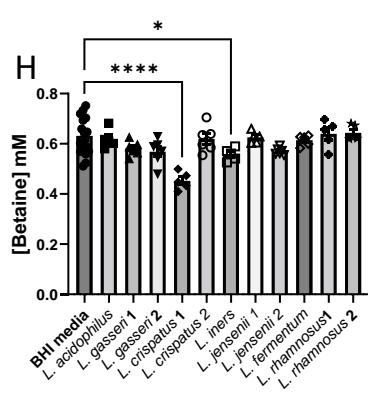

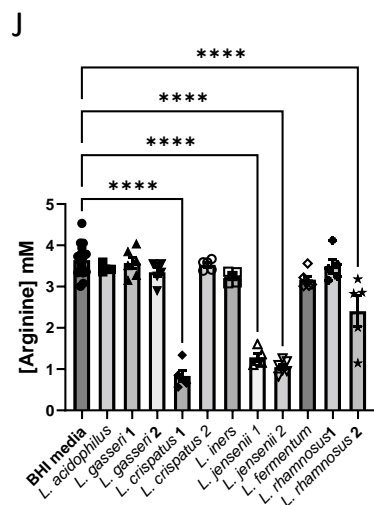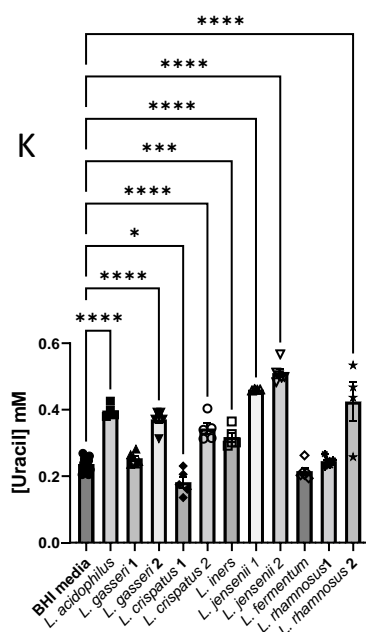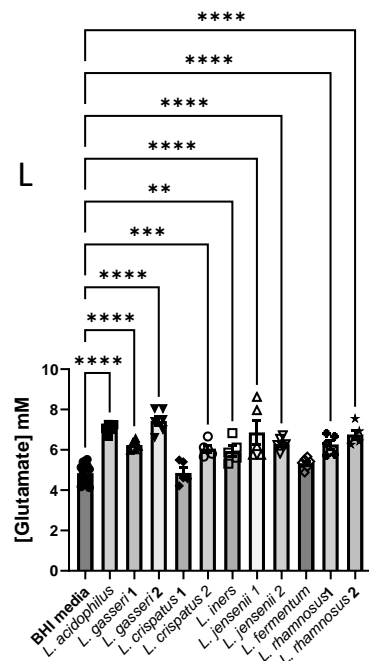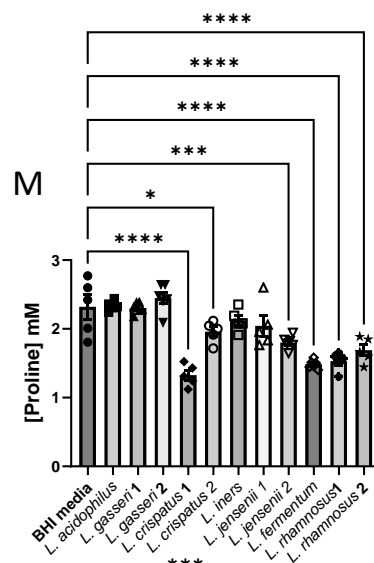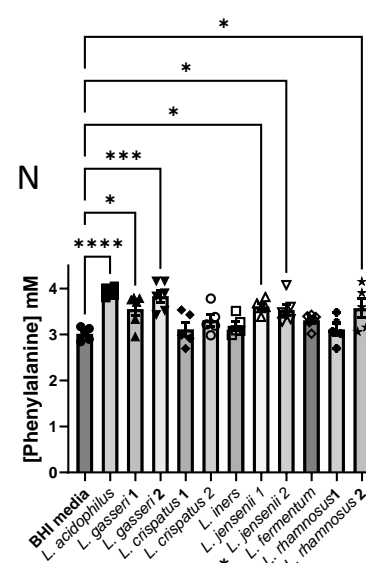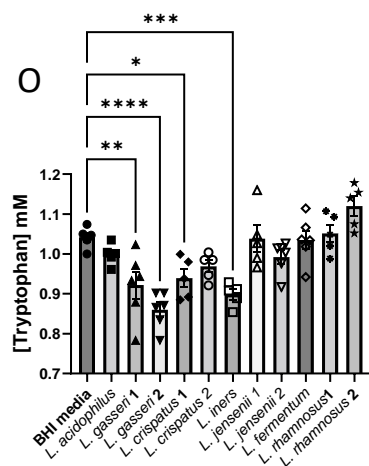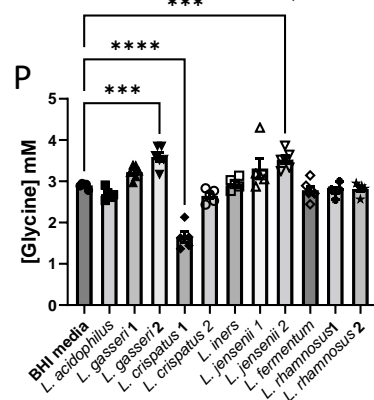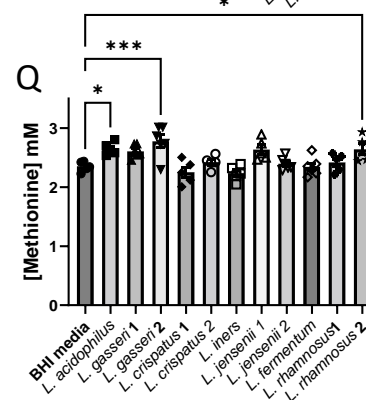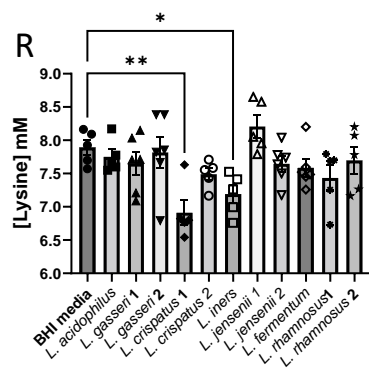

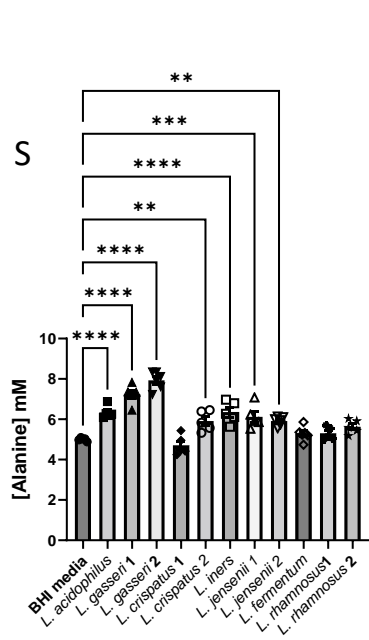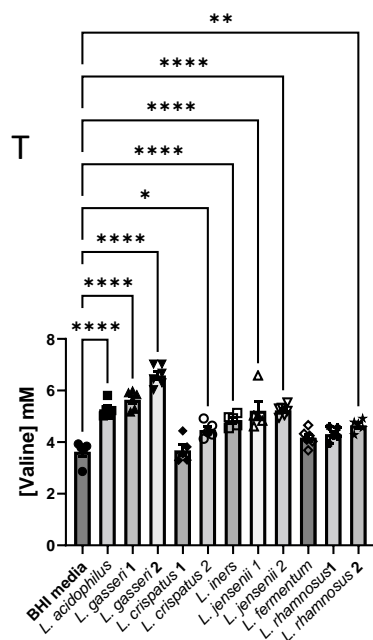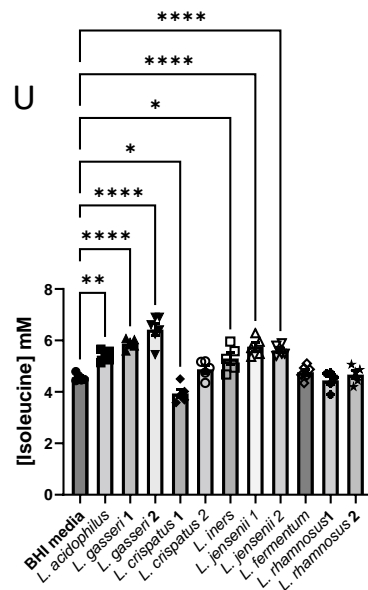

Supplement: FIG S3 [file msphere.00166-22-s0004.pdf]

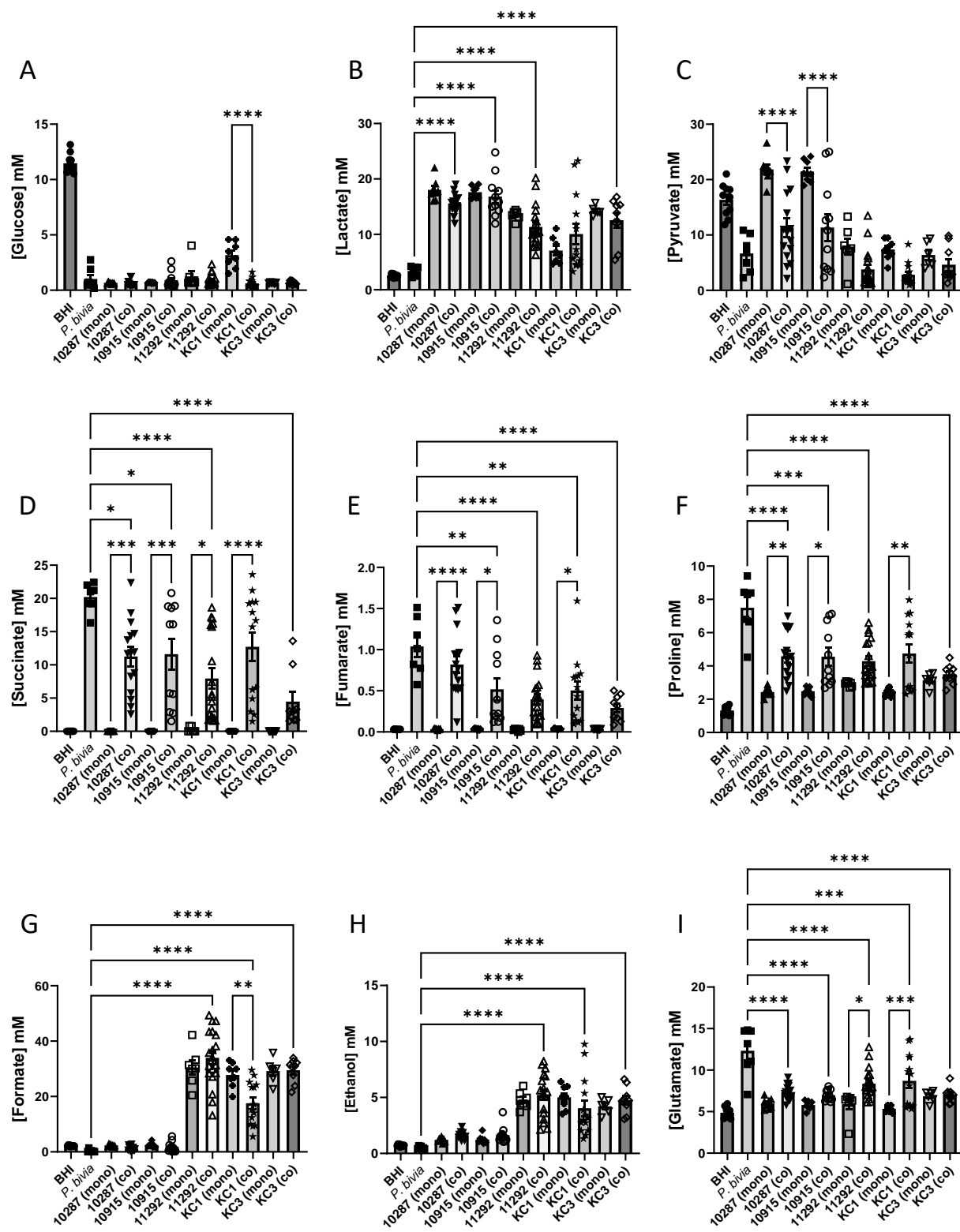

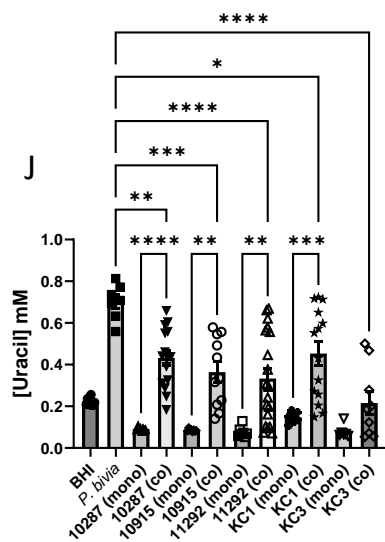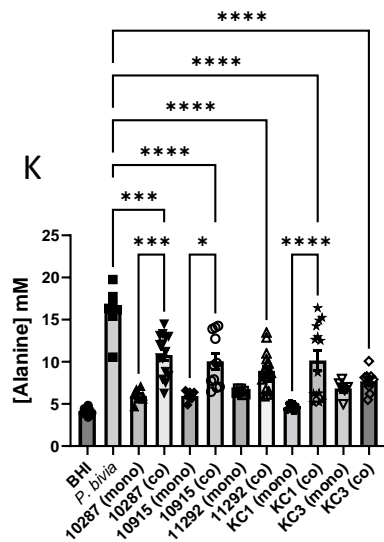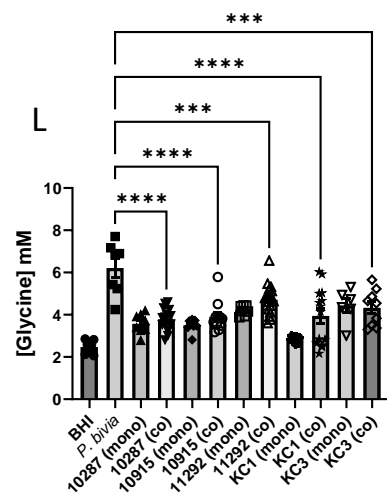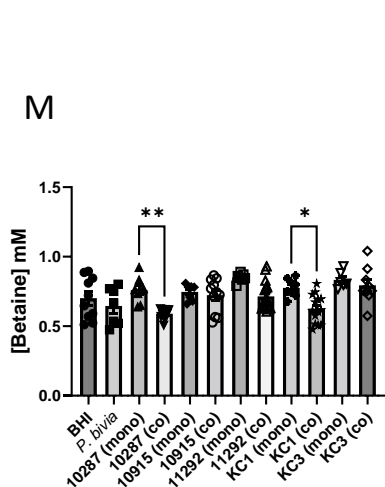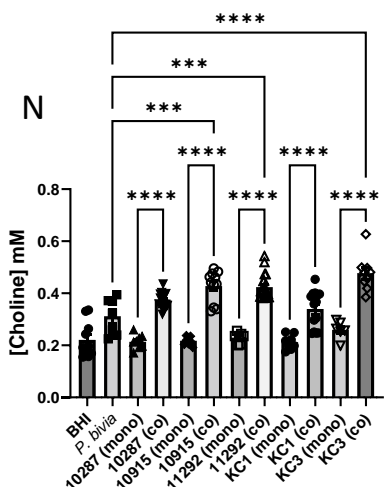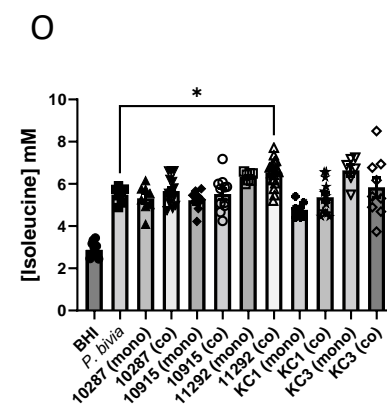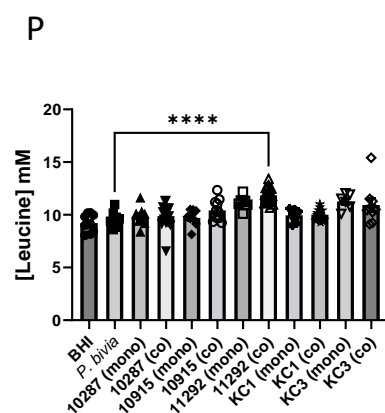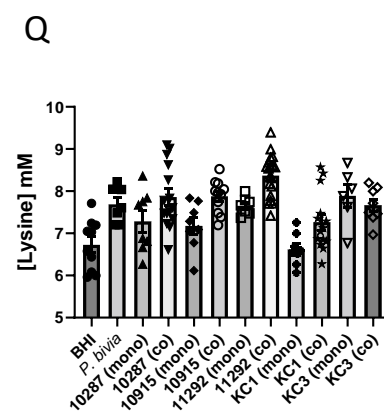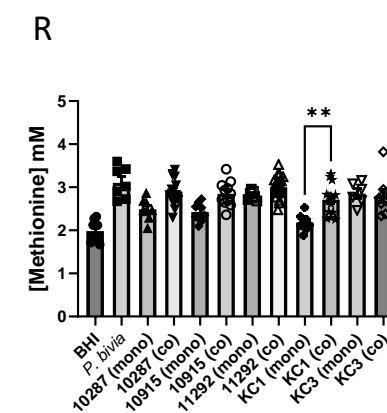

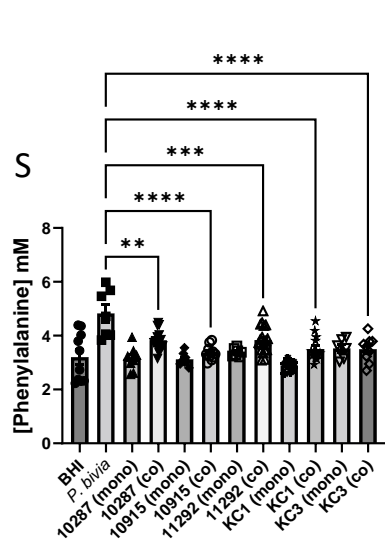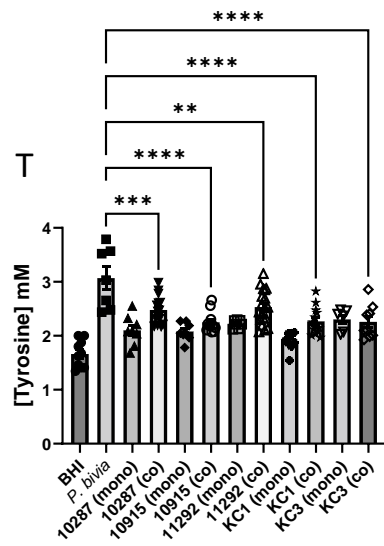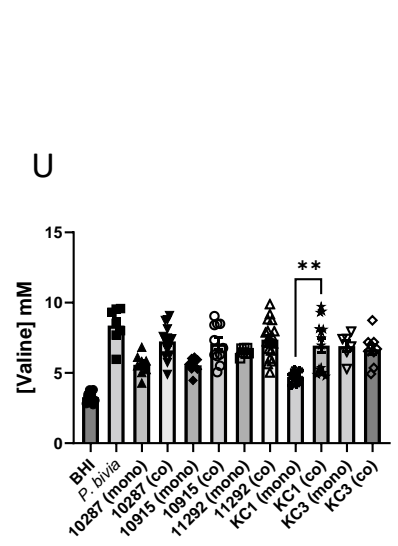

Supplement: FIG S6 [file msphere.00166-22-s0007.pdf]

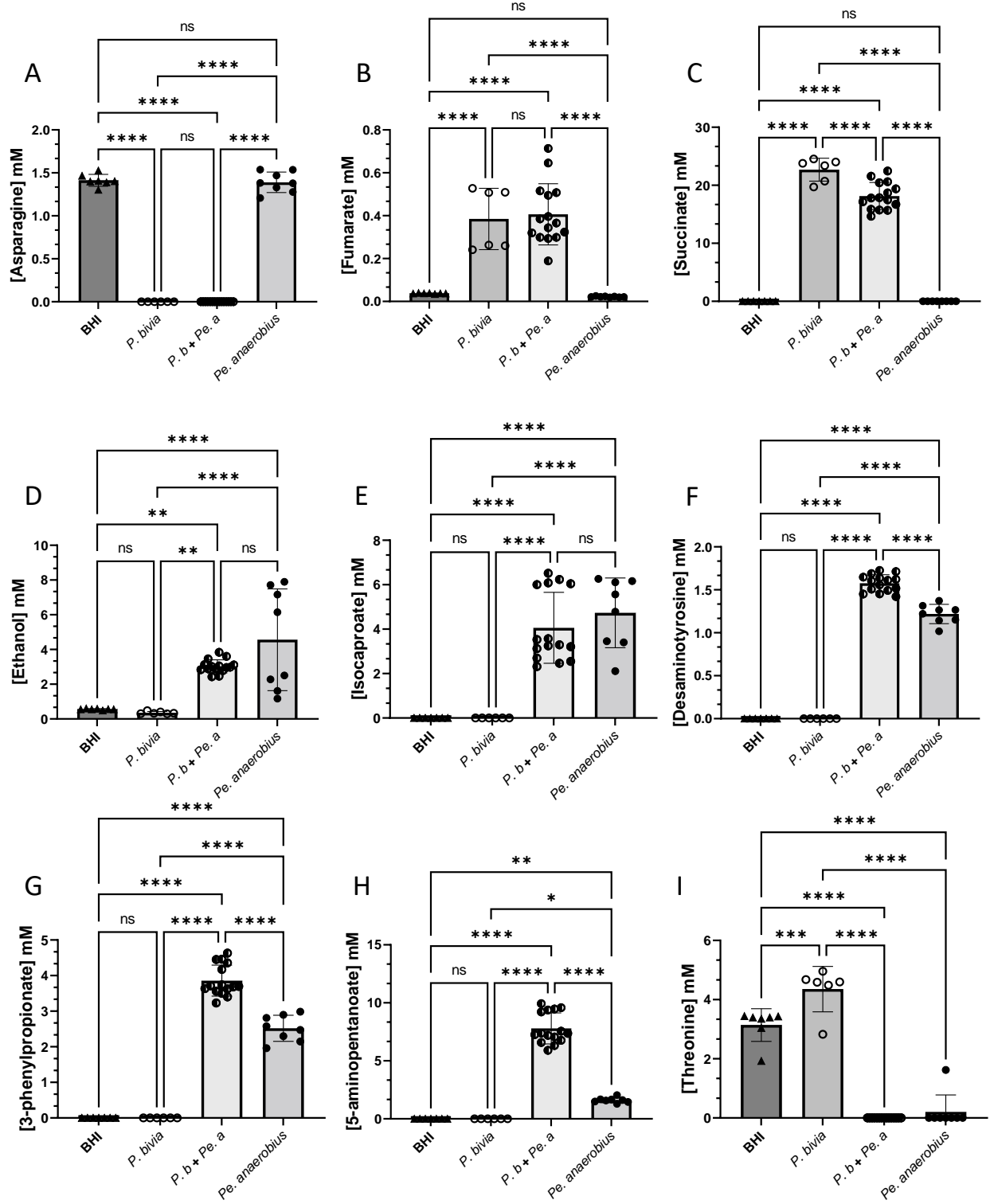

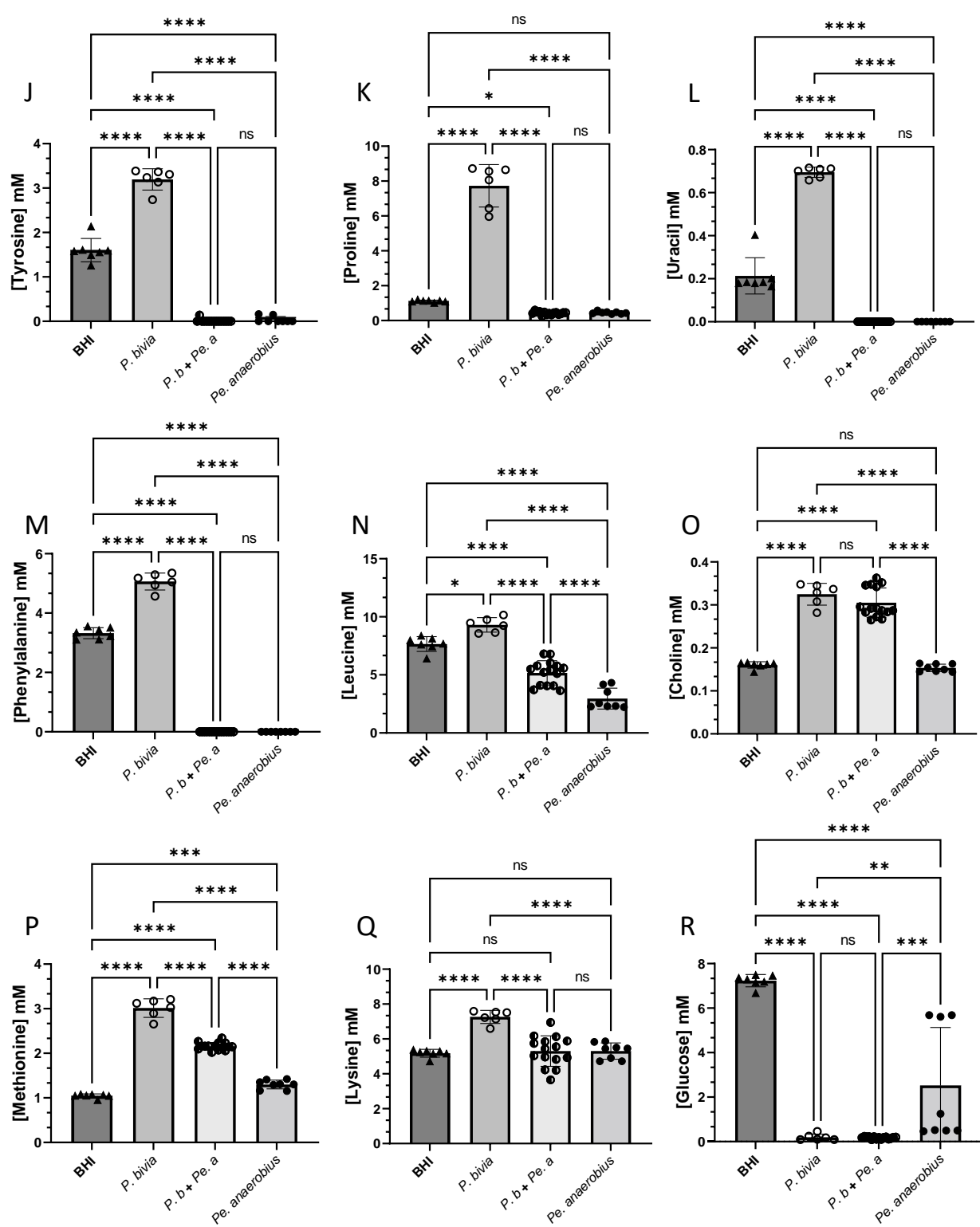

Supplement: FIG S4 [file msphere.00166-22-s0005.pdf]

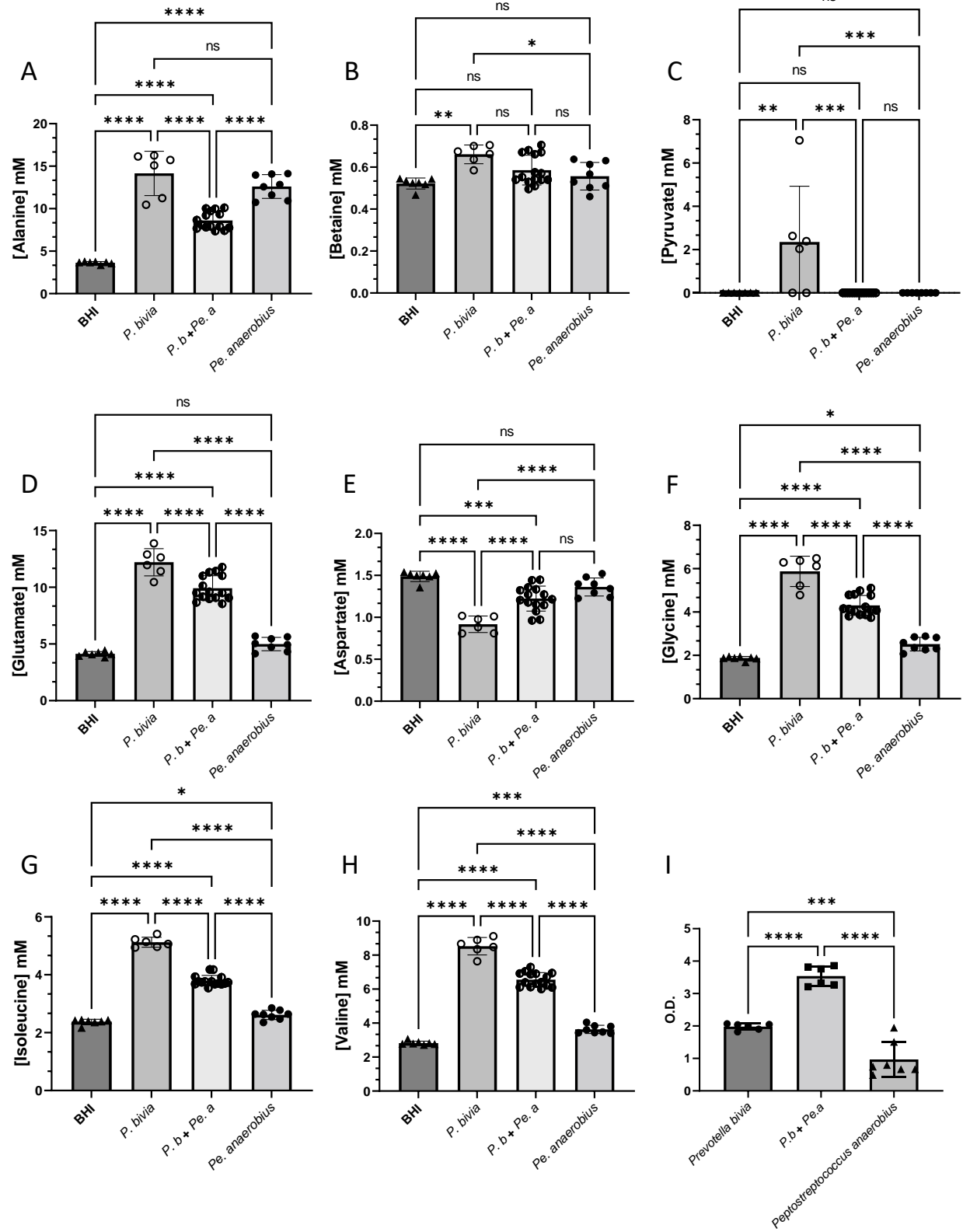

Supplement: FIG S5 [file msphere.00166-22-s0006.pdf]

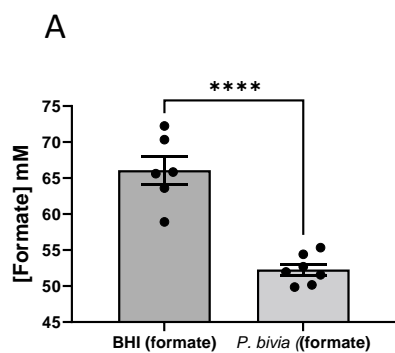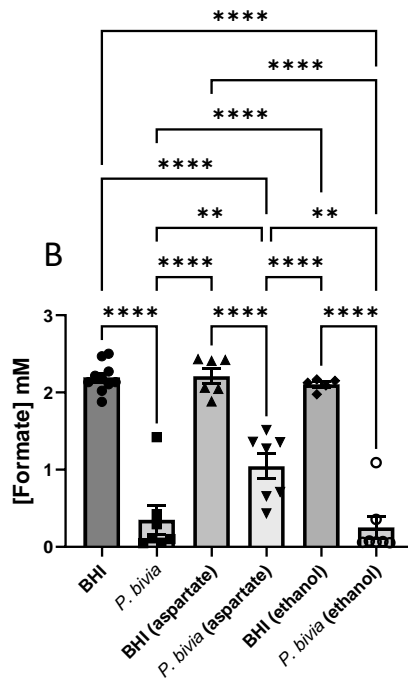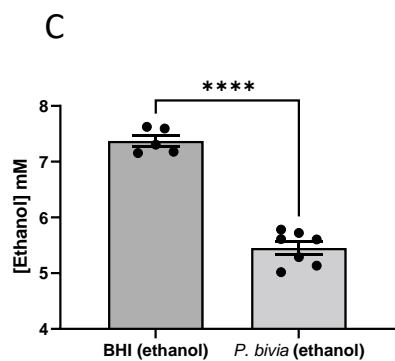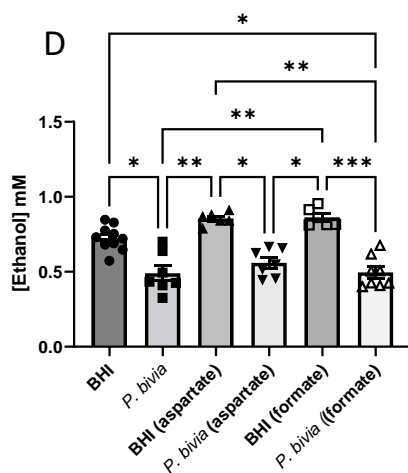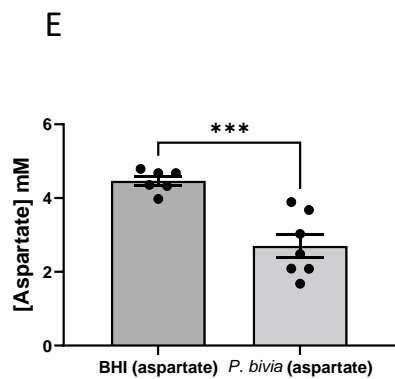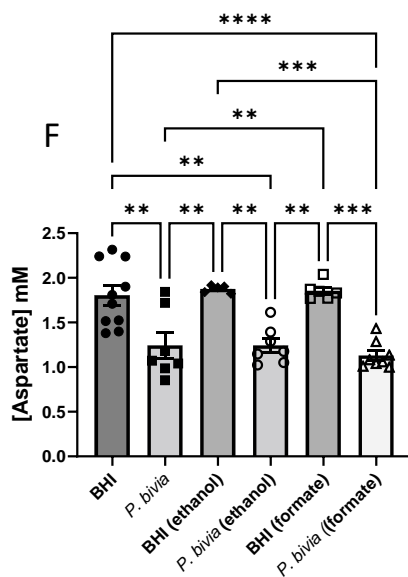

Supplement: FIG S7 [file msphere.00166-22-s0008.pdf]
