# Supplementary material for: Nuclear Magnetic Resonance Metabolomics of Symbioses between Bacterial Vaginosis-Associated Bacteria
Source: mSphere. 2022 May 2;7(3):e00166-22. doi: 10.1128/msphere.00166-22 (PMC9241533; doi:10.1128/msphere.00166-22)
Supplement: TABLE S1 [file msphere.00166-22-s0002.pdf]

Metabolite	CVF swab range	CVF swab mean	Fresh BHI	Spent BHI range
Acetate	0 – 0.049	0.019 ± 0.001	5.71	6.76 – 69.81
Alanine	0 – 0.519	0.103 ± 0.009	4.18	4.07 - 14.87
Arginine	-	0.000 ± 0.000	3.44	0.66 – 3.57
Asparagine	0 – 0.280	0.031 ± 0.005	1.47	0.44 – 2.16
Aspartate	0 – 0.375	0.050 ± 0.007	1.63	1.23 – 2.87
Betaine	0.006 – 0.051	0.012 ± 0.008	0.57	0.44 – 0.64
Cadaverine	0 – 0.744	0.036 ± 0.010	-	-
Choline	0 – 0.632	0.026 ± 0.007	0.19	0.15 – 0.36
Ethanol	0.146 – 0.259	0.219 ± 0.013	0.77	0.79 - 8.64
Formate	0.013 – 0.353	0.052 ± 0.005	2.24	0.66 – 33.73
Fumarate	-	0.000 ± 0.000	0.03	0.03 – 1.97
Glucose	0 – 1.80	0.195 ± 0.029	10.73	0.19 – 9.59
Glutamate	0 – 1.174	0.279 ± 0.025	4.76	4.17 – 9.21
Glutamine	0 – 0.500	0.061 ± 0.007	-	-
Glycine	-	0.000 ± 0.000	2.46	1.64 – 4.69
Isoleucine	0 – 0.323	0.055 ± 0.006	2.79	3.40 – 6.42
Lactate	0.075 – 15.21	3.412 ± 0.290	2.61	2.04 – 48.39
Leucine	0 – 0.679	0.135 ± 0.014	9.22	3.39 – 11.01
Lysine	0 – 0.339	0.039 ± 0.005	6.63	6.12 – 8.21
Maltose	0 – 1.278	0.246 ± 0.029	-	-
Methionine	0 – 0.164	0.025 ± 0.003	1.88	1.30 – 2.78
Ornithine	-	0.000 ± 0.000	0.00	0.00 – 3.53
Phenylalanine	0 – 0.236	0.045 ± 0.005	2.56	0.22 – 3.83
Proline	-	0.000 ± 0.000	1.33	0.59 – 6.04
Putrescine	0 – 0.420	0.015 ± 0.005	-	-
Pyruvate	0 – 0.062	0.008 ± 0.002	18.45	0.97 – 21.60
Succinate	0 – 2.674	0.174 ± 0.043	0.06	0 – 17.84
Taurine	0 – 0.549	0.132 ± 0.011	-	-
Threonine	0 – 0.472	0.069 ± 0.009	2.24	0.00 – 3.19
Tryptophan	-	0.000 ± 0.000	10.4	0.85 – 1.12
Tyrosine	0 – 0.214	0.039 ± 0.004	1.54	0.20 – 2.57
Tyramine	0 – 0.225	0.007 ± 0.003	-	-
Uracil	0 - 0.033	0.002 ± 0.001	0.23	0.03 – 0.63
Valine	0 – 0.358	0.069 ± 0.007	3.04	3.68 – 7.10
